# Tree-ring stable isotopes from the European Alps reveal long-term summer drying over the Holocene

**DOI:** 10.1126/sciadv.adr4161

**Published:** 2025-04-04

**Authors:** Tito Arosio, Markus Leuenberger, Kurt Nicolussi, Jan Esper, Paul J. Krusic, Tatiana Bebchuk, Willy Tegel, Albert Hafner, Alexander Kirdyanov, Christian Schlüchter, Frederick Reinig, Francesco Muschitiello, Ulf Büntgen

**Affiliations:** ^1^ Department of Geography, University of Cambridge, Cambridge CB2 3EN, UK.; ^2^ Climate and Environmental Physics, Physics Institute, University of Bern, 3012 Bern, Switzerland.; ^3^ Oeschger Centre for Climate Change Research, University of Bern, 3012 Bern, Switzerland.; ^4^ Department of Geography, University of Innsbruck, 6020 Innsbruck, Austria.; ^5^ Department of Geography, Johannes Gutenberg University, Mainz, Germany.; ^6^ Global Change Research Centre (CzechGlobe), 603 00 Brno, Czech Republic.; ^7^ Institute of Forest Sciences, Albert-Ludwigs-University Freiburg, Freiburg, Germany.; ^8^ Amt für Archäologie, Kanton Thurgau, Frauenfeld, Switzerland.; ^9^ Institute of Archaeological Sciences, University of Bern, Mittelstrasse 43, 3012 Bern, Switzerland.; ^10^ Sukachev Institute of Forest SB RAS, 660036 Krasnoyarsk, Russian Federation.; ^11^ Institute of Geological Sciences, University of Bern, 3012 Bern, Switzerland.; ^12^ Department of Geography, Faculty of Science, Masaryk University, 613 00 Brno, Czech Republic.

## Abstract

Here, we use 7437 stable oxygen (δ^18^O) isotope ratios extracted from 192 living and relict Alpine trees to reconstruct trends and extremes in European summer hydroclimate from 8980 before the present to 2014 Common Era. Our continuous tree-ring δ^18^O record reveals a significant long-term drying trend over much of the Holocene (*P* < 0.001), which is in line with orbital forcing and independent evidence from proxy reconstructions and model simulations. Wetter conditions in the early-to-mid Holocene coincide with the African Humid Period, whereas the most severe summer droughts of the past 9000 years are found during the Little Ice Age in the 18th and 19th centuries Common Era. We suggest that much of Europe was not only warmer but also wetter during most of the preindustrial Holocene, which implies a close relationship between insolation changes and long-term hydroclimate trends that likely affected natural and societal systems across a wide range of spatiotemporal scales.

## Teaser

Tree-ring stable isotopes from the European Alps reveal long-term summer drying over the Holocene in line with orbital forcing.

## INTRODUCTION

The recent anthropogenic amplification of global warming and associated drought extremes, together with the disruption of ecological and societal systems, has generated much scientific attention (*1*–*4*). Since reliable meteorological measurements are typically restricted to the 20th century, paleoclimate reconstructions are needed to contextualize recent climate changes against past ranges (*5*). However, our proxy-based understanding of natural hydroclimate variability is limited in space and time (*6*), and state-of-the-art Earth system models are still facing challenges to simulating the full spectrum of interannual to multimillennial climate variability over the Holocene (*7*). At the same time, those proxy archives that are sufficiently long, such as ice cores, lake sediments, pollen profiles, and speleothem records, usually lack signal strength on year-to-year and decadal timescales (*8*), whereas shorter tree ring–based climate reconstructions are usually most skillful at higher-frequency domains (*9*). Despite these limitations that also contribute to the confusion about a possible “Holocene temperature conundrum” (*10*), tree-ring stable isotopes (TRSIs) have been demonstrated to reveal hydroclimate variability on different timescales (*9*).

In contrast to more traditional “growth-dependent” tree-ring width (TRW) and wood density parameters, TRSI measurements are considered “growth independent” and therefore able to capture environmental variation well beyond the segment length of individual wood samples (*9*, *11*) . Similar to isotopic ratios in many other terrestrial and marine proxy archives, tree-ring stable oxygen isotopes (δ^18^O) from cellulose primarily reflect the physical state of a tree’s atmospheric and hydrogeological resources (*9*), whereas plant physiological processes of isotopic fractionation, age-related behavior, and issues of sample size are less important (*12*). Empirical evidence for the preservation of multimillennial-long climate trends in TRSI is, however, restricted to three studies in central Europe (*2*), Japan (*13*), and monsoon Asia (*14*), each of them calling for the development of TRSI datasets to advance our understanding of past hydroclimate changes.

**Fig. 1.**
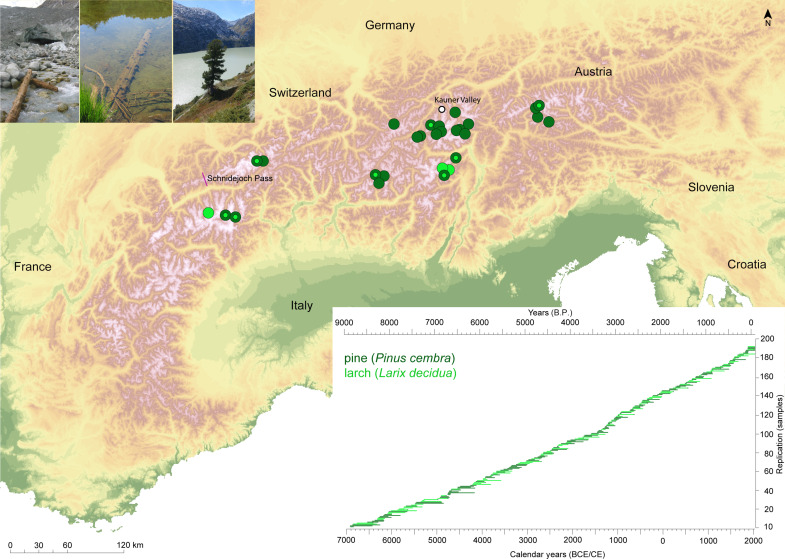
Spatial and temporal distribution of living and relict wood samples. Location of high-elevation sampling sites across the European Alps, with dark and light green referring to larch (*L. decidua* Mill.) and pine (*P. cembra* L.) materials. Top-left inset shows examples of living and relict wood sources, whereas the bottom-right inset refers to the temporal distribution of 82 larch and 110 pine samples over the past 9000 years. Image credit: K.N.

Here, we present 7437 individual δ^18^O values from the α-cellulose of 82 larch (*Larix decidua* Mill.) and 110 pine (*Pinus cembra* L.) trees from high-elevation sites in the Austrian, Italian, and Swiss Alps (Fig. 1). Covering the past 9 millennia (*15*), the continuous TRSI chronology has a 5-year resolution and was developed to preserve information about interannual to multimillennial timescales (fig. S1) (*16*), with age-related δ^18^O trends removed in the first 130 years of cambial growth (fig. S2). The final TRSI chronology consists of raw δ^18^O ratios over the past 6000 years and of TRSI means adjusted for site-specific biases during the earlier portion of the dataset. Outlier trees were corrected by subtracting half the offset from the chronology mean during overlapping periods, thereby preserving long-term trends and reducing short-term fluctuations (fig. S1).

## RESULTS

### Hydroclimate interpretation

**Fig. 2.**
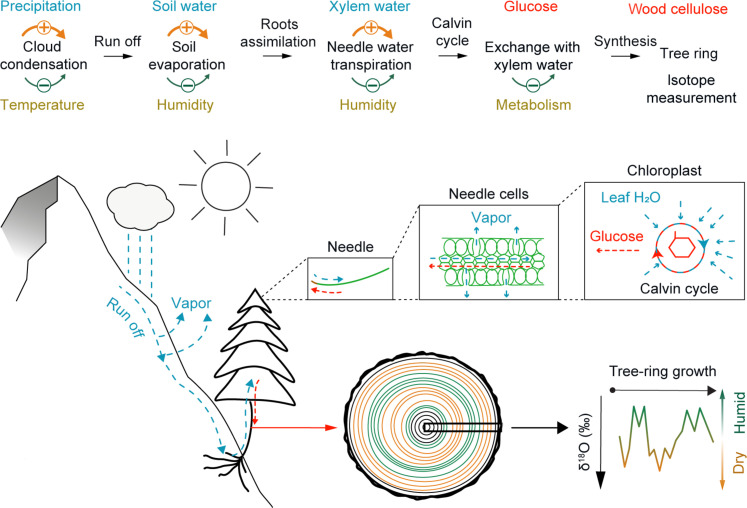
Biotic and abiotic drivers of oxygen TRSIs. Oxygen isotopes (δ^18^O) in precipitation are subject to the Rayleigh distillation, where they are influenced by condensation temperature, whereas δ^18^O in soil water is influenced by evaporation, which is sensitive to humidity. The δ^18^O composition of leaf water is enriched by transpiration, which is mainly influenced by relative atmospheric humidity, and imprinted in the synthesis of glucose through the Calvin cycle under constant fractionation. Since glucose forms cellulose, tree-ring δ^18^O contains a distinct hydroclimate signal.

The hydroclimate signal in our TRSI record is predominantly driven by soil-infiltrating precipitation and subsequent evaporation (Fig. 2) (*17*). Soil water is transported to the leaves without fractionation where it is subject to transpiration (*18*). Since lighter isotopes evaporate faster, evaporation and transpiration both enrich the leaf with heavier isotopes. The degree of evaporation and transpiration, and, thus, the enrichment of heavier isotopes in leaf water, is influenced by relative humidity (*18*). Leaf water is incorporated in glucose under constant biological fractionation (*18*). Since glucose is used to synthesize cellulose under a second-order fractionation process, the isotopic composition of wood cellulose equals that of leaf water (*12*). Increased (decreased) precipitation totals and/or lower (higher) mean temperatures decrease (increase) evapotranspiration and lead to lower (higher) tree-ring δ^18^O values.

Our TRSI record reflects year-to-year and longer-term changes in summer hydroclimate due to the predominance of convective moisture that originates from either central or southern Europe (*19*). Long-term trends in the reconstructed δ^18^O values can be attributed to the effect of temperature on precipitation isotopes, which have been obtained independently from speleothems and ice cores (*20*, *21*). While other proxy archives exhibit a negative δ^18^O trend associated with long-term temperature cooling (*20*, *21*), our tree-ring δ^18^O reconstruction reveals a contrasting positive trend (fig. S1G). This inverse relationship suggests that the δ^18^O signal in tree rings primarily represents hydroclimate rather than temperature effect on precipitation isotopes, a finding further validated by our correlation analysis (fig. S3). While short-term correlations show a positive relationship between temperature and tree-ring δ^18^O, the long-term trend of our TRSI chronology agrees with temperature reconstructions from ice cores and speleothems (fig. S4). This apparent contradiction is resolved by recognizing a long-term increase in tree-ring δ^18^O that is primarily driven by long-term drying, as evidenced by leaf water δ^18^O enrichment. This occurs despite the decrease in precipitation δ^18^O over the Holocene in this region. Explained by a simple mechanical model of isotopic fractionation (Fig. 2), this relationship is considered stable over time.

### Hydroclimate reconstruction

**Fig. 3.**
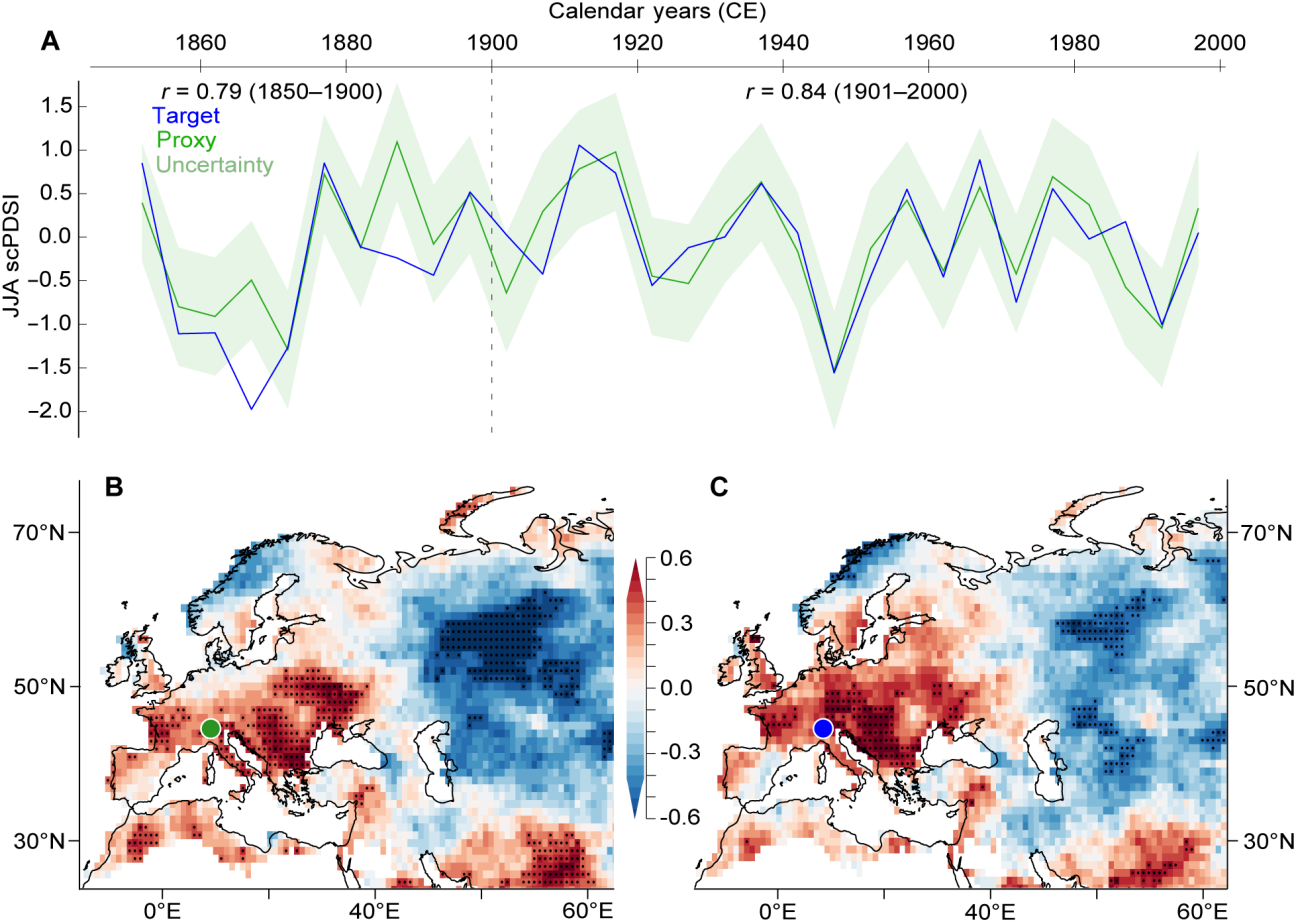
Proxy-target relationship. (**A**) Behavior of the measured (blue) and reconstructed (green) scPDSI (*23*) averaged over JJA at a 5-year resolution. Correlation coefficients *r* of 0.84 and 0.76 over the two independent calibration (1901–2000) and verification (1850–1900) periods are both highly significant (*P* < 0.001). Our δ^18^O TRSI-based reconstruction has a mean square error of 0.36 (green shading). (**B**) Spatial correlation maps (0.5° by 0.5°) between our Alpine summer hydroclimate reconstruction (green dot) computed against gridded, European-wide scPDSI target data over the 20th century. (**C**) Similar to (B) but using an Alpine subset of the measured JJA scPDSI instead of the reconstructed JJA scPDSI. The dots indicate the significance of correlation (*P* < 0.001).

Comparison between our final δ^18^O chronology and a regional subset of the self-calibrated Palmer drought severity index (scPDSI) (46° to 47°N and 7° to 12°E) (*22*) averaged over June to August (JJA) reveals a significant positive correlation coefficient of 0.69 (*P* < 0.001; 1901–2000) (figs. S3 and S5). The correlation further increases to 0.84 (*P* < 0.001; 1901–2000) when using an Alpine subset of the scPDSI (*23*). While linear regression over 1901–2000 was used for calibration (Fig. 3A), the 1850–1900 period was used for verification (*r* = 0.76, *P* < 0.01). A slight decrease in proxy-target agreement during the 19th century likely relates to a decline in the quality of the scPDSI target rather than a weakening of the δ^18^O proxy, which is characterized by constant sample quality and quantity back in time (fig. S1B). The distinct summer hydroclimate signal in our δ^18^O chronology corroborates previous, although much shorter, reconstructions that also used TRSI data as predictors (*24*, *25*).

European-wide spatial correlation maps between the reconstructed and measured JJA scPDSI visualize the signal strength of our record over large parts of central Europe, Italy, and the Balkan Peninsula (Fig. 3B). A similar pattern from the measured Alpine scPDSI (*23*) implies humid conditions in the surrounding lowlands of Alpine arc enhance convective moisture transport into the mountains. This assumption is reinforced by negative correlations with potential summer evapotranspiration across the Alpine arc and beyond (fig. S6).

**Fig. 4.**
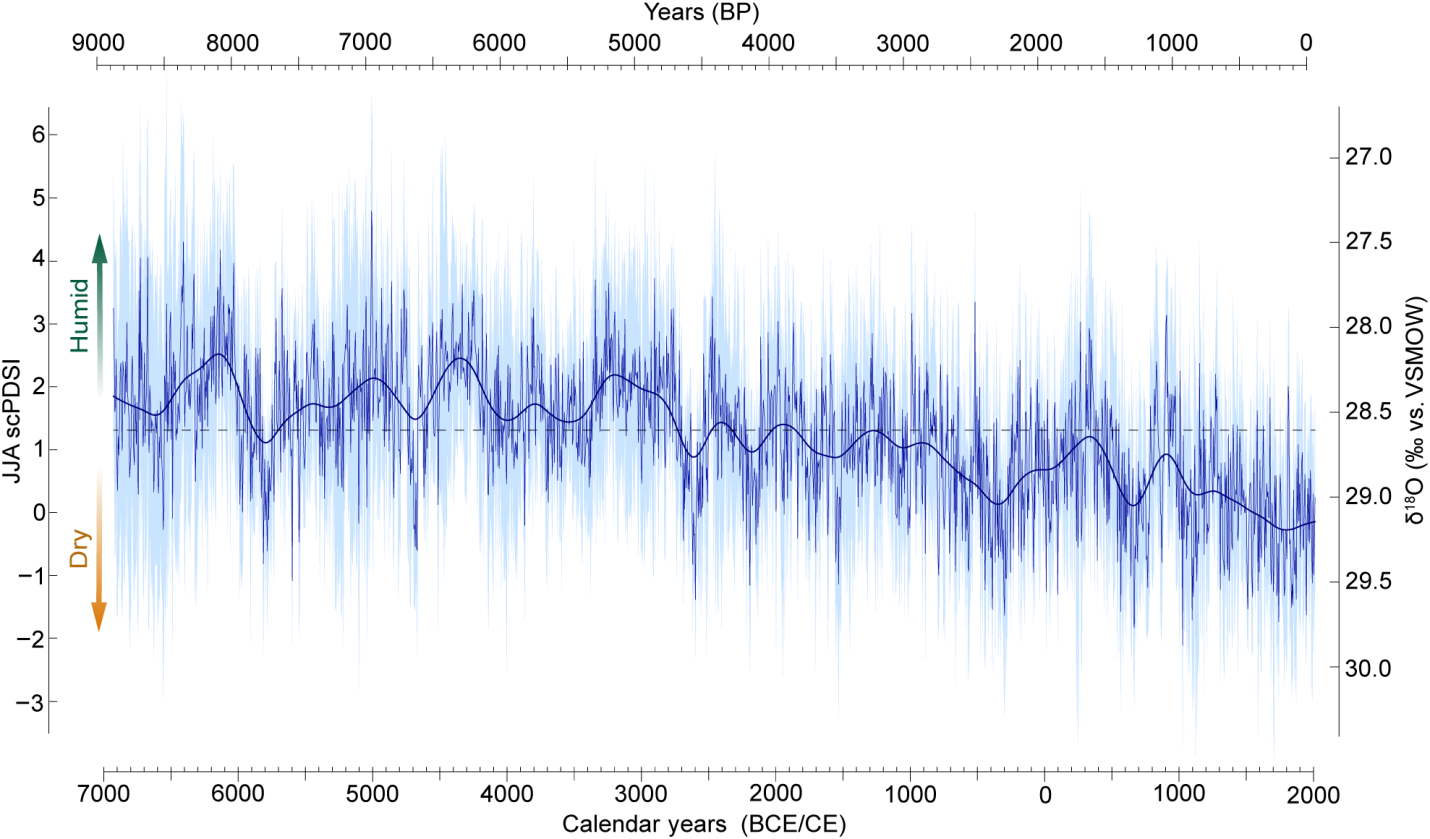
Alpine summer hydroclimate reconstruction. Our δ^18^O TRSI-based JJA scPDSI reconstruction from the European Alps that covers the past 9000 years at a 5-year resolution (dark blue), together with its 500-year low-pass filter (smoothed line) and uncertainty range (light blue). The dashed horizontal line refers to the reconstructed long-term mean. VSMOW, Vienna Standard Mean Ocean Water.

When considering the past 9000 years before the present (B.P.) (i.e., before 1950 CE), our TRSI-based Alpine summer scPDSI reconstruction exhibits a significant long-term drying trend (*P* ≤ 0.001, −0.226 JJA scPDSI/1000 years), with substantial interannual to multicentennial variability superimposed (Fig. 4). Most notable is the extent of reconstructed humidity during the early Holocene, which is followed by a marked drop toward drier conditions between approximately 8000 and 7500 B.P. The mid-Holocene also contains phases of distinct humidity around 6300 and 5000 B.P., while long-term drying is most distinct during the late Holocene. Episodes of marked summer drought occurred around 2300 and 1400 B.P., and the driest summers coincided with the Little Ice Age (LIA) in the 18th and 19th centuries CE (table S1 and fig. S7).

**Fig. 5.**
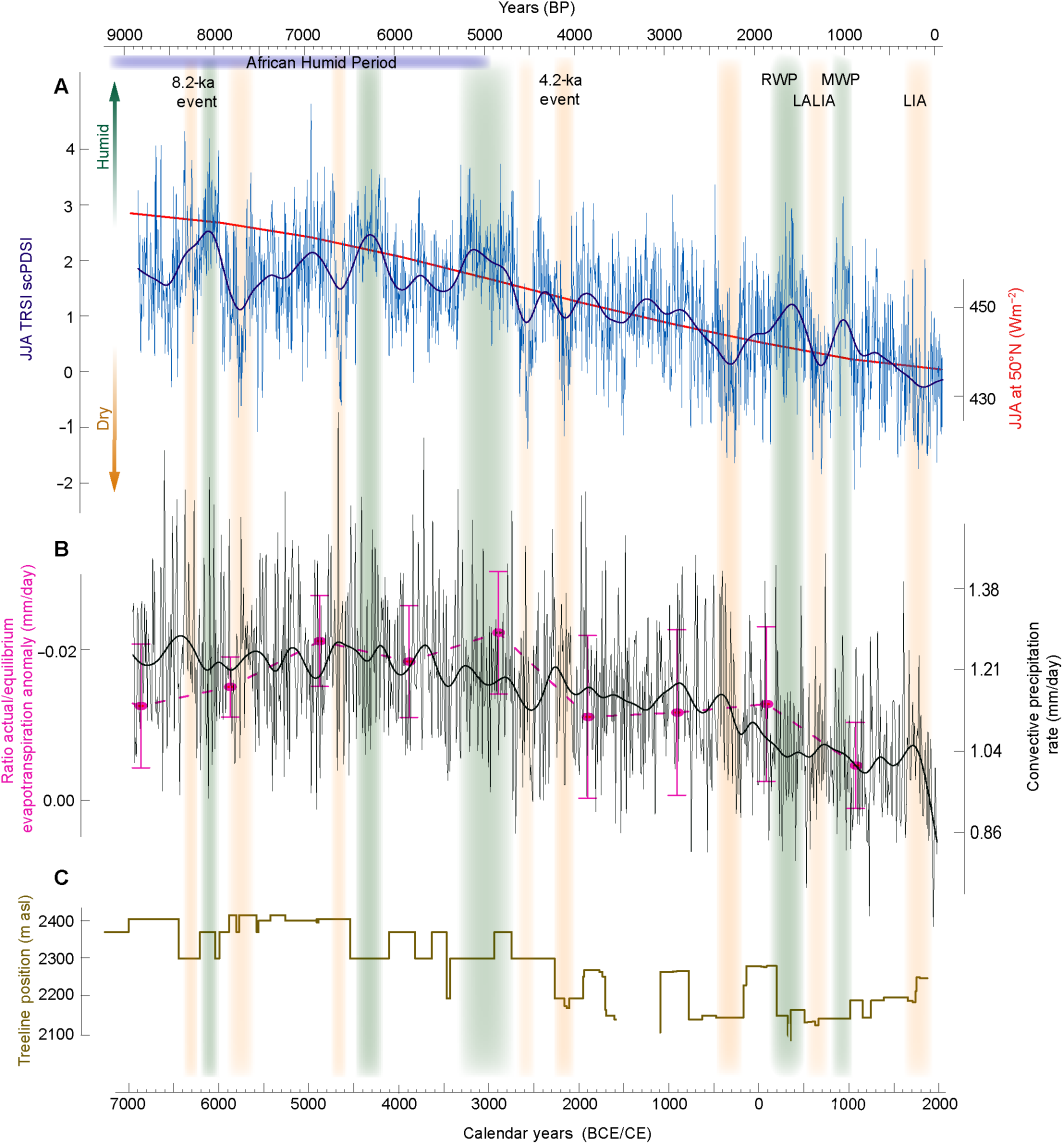
Alpine climate and environmental changes over the Holocene. At the top, climatological phases shown are characteristic of the past 9000 years in central Europe and the Alpine arc, including the African Humid Period, the 8.2- and 4.2-ka events, the Roman Warm Period (RWP), the Medieval Warm Period (MWP), the Late Antique LIA (LALIA), and the LIA. (**A**) Our δ^18^O TRSI-based JJA scPDSI reconstruction from the European Alps (dark blue) and its 500-year low-pass filter (smoothed line), together with the accumulated Holocene orbital forcing for JJA at the mid-northern latitudes (50°N) (*78*, *79*) (red line). Vertical bars highlight wet (light green) and dry (light red) episodes that deviate from the long-term trend of our reconstruction. (**B**) Mean annual ratio of the equilibrium evapotranspiration anomaly (in millimeters per day) reconstructed for the Holocene (pink) for the grid 46° to 47°N and 7.55° to 12.25°E (*76*). Annual mean value of the convective precipitation (black) simulated with the fully forced TraCE-21K for the grid 46° to 47°N and 7.55° to 12.25°E (*77*). (**C**) Estimated changes in the position of the upper treeline in the Austrian Alps (*27*). asl, above sea level.

The observed long-term drying parallels a negative trend of orbital forcing over most of the Holocene (Fig. 5A) (*26*), which is also in line with a Holocene-long cooling trend (*20*, *27*). The long-term behavior of our reconstruction is, however, not directly caused by the temperature signal in the δ^18^O of precipitation. Instead, this trend in TRSI is consistent with a change under hydroclimate conditions rather than a direct change in the δ^18^O of precipitation captured by speleothems (*20*) (fig. S4). The gradual drying trend in our record agrees with climate model simulations on both the convective precipitation rate (Fig. 5B) and the total precipitation rate (fig. S8). Overall, wetter conditions in the early Holocene coincide with the African Humid Period (*28*). Previous multiproxy reconstructions from the Mediterranean region also suggest a wetter and warmer first half of the Holocene (*29*), in line with reduced glacier extents (*30*) and higher treeline positions across the Alpine arc (Fig. 5C). Enhanced solar insolation during mid-Holocene boreal summers likely strengthened convective moisture transport over Europe (*31*), which had a stronger impact on drier summers (*32*). As the mid-Holocene progressed, decreased summer insolation contributed to drier conditions not only in the Alps but also across Europe, which are reflected in both model simulations and proxy reconstructions (Fig. 5B) (*32*). The long-term drying of our reconstruction further agrees with a pollen-based reconstruction of evapotranspiration (fig. S9), as well as with model-based convective precipitation rates (Fig. 5B and fig. S8). Prolonged humidity around 8100 B.P. was preceded by a drier phase around the 8.2-ka event (*33*), during which temperatures in central Europe dropped sharply (*34*), Alpine glaciers advanced (*30*) and lake levels on the Swiss Plateau were higher (*35*). Wetter conditions have also been reported for central Italy from around 8300 to 7900 B.P. (*36*), which corroborates increased humidity over Greenland during the same period (*37*). Another noticeable wet period in our reconstruction around 5000 B.P. matches with reports from marine sediments and speleothem records from the Mediterranean (*29*) and central Italy (*36*) and agrees with independent evidence from Alpine speleothems that show a humid phase in the European Alps between around 5300 and 4700 B.P. (*38*). Increased mid-Holocene humidity was likely driven by the northward Intertropical Convergence Zone shift steering enhanced northern subtropical and reduced tropical precipitation (*39*, *40*) (fig. S10, A to C). This migration was likely accompanied by a contraction of the subtropical high-pressure belt and altered regional moisture dynamics (*41*). Modeled pressure patterns indicate diminished North Atlantic westerlies (*39*) (fig. S10D), with central European moisture originating from the Mediterranean, where elevated sea surface temperatures likely amplified evaporation and moisture supply during this period (*41*, *42*).

For the CE, we found evidence for a humid period in the 4th and 5th centuries CE (Fig. 5), when warmer and wetter conditions have been reported for Europe from a range of proxy archives (*43*). Drying during the 6th century CE overlaps with the Late Antique LIA (*44*), which has previously been described as particularly dry in central Europe (*2*, *45*). Our reconstruction suggests a relatively humid Medieval Warm Period from around 900 to 1200 CE (*46*). The driest reconstructed summers of the past 9000 years coincide with the coldest phase of the late Holocene (*46*), i.e., the peak of the LIA in the 18th and 19th centuries CE.

## DISCUSSION

Although the high spatiotemporal resolution of our TRSI-based summer hydroclimate reconstruction allows for comparison with archaeological and historical evidence (*47*), detailed interdisciplinary investigations into the entanglements between climate variability and human history are outside the scope of this work. Prominent examples, however, include the relatively stable hydroclimate conditions between around 7600 and 6900 B.P. during which the first agricultural communities emerged and consolidated in central Europe, known as Linear Pottery culture (LBK) (*48*), or the stable phase from ~6200 to 5500 B.P. during which new agricultural land was established along the shorelines of the pre-Alpine lakes (fig. S11). Future research will hopefully offer additional insights into the way that hydroclimate has affected the functioning and productivity of pastoral and agricultural systems and the economic and political structures of societies. High-resolution paleoclimate data, qualitative analysis, and consideration of both structural and contextual variables that define a society at the time of climate and environmental anomalies are needed to unravel direct and indirect causal connections between hydroclimate changes and possible socioeconomic, political, and cultural responses.

Our data indicate significant long-term drying trend over much of the Holocene and that climate in Europe was not only warmer but also wetter during most of the preindustrial Holocene, with the presence of extremes. This central European summer hydroclimate reconstruction may facilitate the establishment of robust relationships between environmental and societal systems (*47*) that could offer insights on how hydroclimate has affected pastoral and agricultural systems and the economic and political structures of societies.

In synthesis, our findings (i) provide a long-term context for evaluating trends and extremes in 21st century hydroclimate, (ii) help constraining the uncertainty of Earth system model simulations, (iii) inspire the development of high-precision climate proxy records for the Holocene, and (iv) stimulate interdisciplinary investigations into the entanglements between environmental and societal systems during the past 9000 years.

## MATERIALS AND METHODS

### Study area and wood samples

Wood samples of deciduous larch (*L. decidua* Mill.) and evergreen cembran pine (*P. cembra* L.) were collected across the central Alps, in the latitudinal range between 46.05° and 47.08° north and the longitudinal range between 7.55° and 12.25° east. All sites are situated in high-elevation climatic subregions (*49*), from the treeline (*15*), at elevations ranging from 1950 to 2300 m above sea level (Fig. 1 and table S1). In these subalpine areas, summer temperature is the primary limiting factor for tree growth (*15*). The Alpine arc receives moisture from multiple sources, including the North Atlantic sector, central Europe, and the Mediterranean basin (*19*, *50*). This moisture results in a precipitation peak in the Alpine region during the summer months.

The TRW measurements of the wood samples were used to develop a 9000-year-long Eastern Alpine Conifer Chronology (EACC) by the Department of Geography of the University of Innsbruck, with a mean Rbar and a mean expressed population signal of 0.26 and 0.89, respectively (*15*). The temporal accuracy and reliability of the EACC were recently verified by the demonstration of several ^14^C (Miyake) events (*51*–*54*). The oldest ^14^C event recorded in the EACC dates back to 7176 BCE. The TRW statistics for each tree are reported in fig. S12 and table S2. Samples with relatively wide rings were selected to provide enough material for the isotope measurements. As described in previous studies (*16*, *55*) and in the publicly available database Alpine Holocene Triple Tree Ring Isotope Record (AHTTRIR) (*56*), all wood samples of the database have a 5-year resolution.

### Stable isotope measurements

The samples underwent cellulose extraction following the procedure detailed in Ziehmer *et al.* (*57*) and were subjected to the triple-isotope analysis (*58*, *59*). We used conventional isotope ratio mass spectrometry (Isoprime 100) coupled with a pyrolysis unit (HEKAtech GmbH, Germany), similar to the temperature conversion/elemental analysis (TC/EA) method (*60*), with an extension for measuring nonexchangeable hydrogen in α-cellulose using the online equilibration method (*58*, *59*). Results are reported in per mil (‰) relative to Vienna Standard Mean Ocean Water (VSMOW) (*61*). Calibration was performed using two-point calibrations with Merck cellulose and IAEA-CH-6 crystalline sugar for carbon and oxygen isotopes. Quality control was assured using Merck and Wei Ming 101 cellulose as well-characterized internal laboratory standard materials. The precision of measurements was ±0.3‰ for oxygen, in line with the methodology described by Loader *et al.* (*58*) and Filot *et al.* (*59*).

### Chronology development

Prior work showed that tree-ring δ^18^O values in this database exhibited age-related trends in the first 100 cambial years, with similar patterns in both larch and pine (*16*). However, the mean cambial age across all samples was 205 years, with a minimum of 1 year and maximum of 725 years. Thus, these age-related trends affected only a minor portion of the data. To eliminate such age effects, we applied a residual species-specific 80-year spline detrending to each tree’s first 130 years while retaining the absolute mean value. This involved subtracting the difference between spline value and the mean values for a given cambial year from the corresponding tree’s δ^18^O value. Before detrending, the correlation between δ^18^O and cambial age was statistically significant (*r* = 0.2, *P* < 0.05). After detrending, this correlation became nonsignificant for both species (*r* > 0.1, *P* > 0.01). Beyond 130 cambial years, δ^18^O values exhibited no significant age dependence (*r* > 0.1, *P* > 0.01) (fig. S2A). These results support prior findings that tree-ring δ^18^O shows minimal age-related trends past the juvenile growth stage (*62*, *63*).

We previously demonstrated that larch and cembran pine do not exhibit species-specific values in δ^18^O (*55*). Here, we show that the species-specific chronologies have a significant positive correlation (*r* = 0.5, *P* < 0.05) in the periods of overlap. In the period from 1900 to 2000, the replication of the two species remains constant, and the correlation becomes even more pronounced, with a factor of 0.7 (*P* < 0.05). This supports the notion that tree-ring δ^18^O values tend to exhibit a similar low-frequency (decadal-scale) signal across different species (*64*) and allows us to combine the two species into a single chronology. During the calibration period, where there is a constant number of trees at three sites, the intersite correlations are computed in addition to the interspecies correlation. The correlation factors among the three sites are 0.8, 0.7, and 0.5 (*P* < 0.05), with two trees per site.

Geographical correction and disturbance tests were performed to ensure the accuracy of the chronology constructed from the tree-ring δ^18^O data. We evaluated several methods, including normalization of all trees and methods proposed by Hangartner *et al.* (*65*) and Nakatsuka *et al.* (*13*). However, it was found that none of these approaches was able to correct for offsets between trees and preserve the long-term trend present in the raw data, mainly due to the lower temporal resolution of datasets for which the methods were developed compared to an annually resolved dataset. The offsets between some trees and the rest are the cause of high values of SD in some periods, such as around 8000 and 6000 B.P. (fig. S1, C and D).

On the basis of this analysis, it was concluded that the raw data itself provided the most representative chronology from 6000 years ago to the present, despite the presence of some outlier trees that were corrected only with tree offset correction (described below). However, in the early Holocene (around 8000 to 9000 B.P.), we found large differences between various sampling sites, resulting in a higher SD in the overlapping time frame between the regions (fig. S1C). To address this issue, we conducted a disturbance test on the chronology value by testing whether the running mean value of 20 years was within the mean value ± SD of the previous 400 years. We found that all the points that deviated from the system were in the early Holocene and at the intersection between different regions. To account for the discrepancies, we adjusted the means of all trees in the time window by the offset values, so that the mean was equal to the mean of the rest of the chronology over the overlap period. Tree populations with a constant offset from the chronology came from only two regions. Corrections were made for the following three periods only: 6200–5700 B.P., 6500–7500 B.P., and 8000–9000 B.P., and were applied only to trees originating from the two regions. The corrections were applied to the overall means within each region and interval, rather than to individual tree measurements. This synchronization of the regional means for the older and younger areas allowed the SD to be reduced in the period where the region overlaps. We validated this correction repeating the disturbance test.

Despite the prior corrections, some individual trees still exhibited an offset compared to the mean distribution across all trees. If unaddressed, then these offsets could influence short and medium-term variance. This offset may be due to microclimate conditions or a slightly different source of water, such as a river, that affects the absolute values of δ^18^O. In some periods (e.g., around 500 CE), the raw data of some trees do not overlap with the ordinary range of the chronology (fig. S1E). Our assumption is that the database variance of the past 200 years (1800–2010 CE) should indicate the maximum range of the tree value compared to the chronology mean value, since, in this period, a climate sensitivity analysis was conducted. Thus, we applied a variant of the method proposed by Nakatsuka *et al.* (*13*), which includes merging the overlapping tree-ring isotope series that can create artificial trends (*13*, *66*). We chose a less stringent method to preserve the long-term trends. The offset trees outside the ordinary range were identified as those with mean exceeding the rolling mean value of the same period plus or minus the SD of the calibration time (1850–2015 CE). The half difference between the mean of the tree values and the mean of the chronology was subtracted for these trees in the overlap period. This operation was repeated in a loop until no tree mean was outside the ordinary range.

On the corrected δ^18^O values, we ran a final analysis similar to that performed in 6000-year-long isotope chronology (*14*), comparing the δ^18^O with latitude, longitude, elevation, and cambial age (figs. S13 to S16). No significant relationship between mean δ^18^O and mean latitude, longitude, cambial age of the samples, and sample replication was detected (figs. S13 to S17).

### Tree-ring isotopes statistics

The mean series length of our dataset is 194 years, with a maximum of 611 and a minimum of 16, making the length of the series extremely variable. With a 5-year resolution, the mean measured sample length is 39 samples, with a maximum of 122 samples and a minimum of 3 samples. The different segment lengths and the nonannual resolution make the application of the traditional tree-ring statistics less informative. The mean interseries correlation is *r* = 0.45, with a maximum of 0.86 and a minimum of 0.10 (fig. S12). It is important to remember that the oxygen isotope series have not been cross dated; instead, the chronology was developed using the TRW-based cross dating.

### Hydroclimate sensitivity

Multiple climate variables spanning 1901–2000 CE were obtained from the Climate Explorer website (https://climexp.knmi.nl/) for the grid 46.03° to 47.05°N and 7.55° to 12.25°E (that includes all the sampling sites), including monthly Climatic Research Unit (CRU) Time-series (TS) 4.07 precipitation, temperature, cloud cover, vapor pressure, and daily temperature range; Standardized Precipitation-Evapotranspiration Index (CSIC SPEI); and scPDSI (*23*). We also used the measurement-based Greater Alpine Region scPDSI as an index of Alpine drought (*23*). All climate variables were interpolated by averaging the data values within 5-year blocks to match the δ^18^O resolution. Monthly climate sensitivity analysis was conducted for each variable over 1901–2000 CE (figs. S3 and S5). Correlations were stronger for summer months. Figure S5 shows the correlation between reverse δ^18^O and each JJA climate parameter, both before and after first-order detrending. Graphical analysis of Alpine scPDSI (*23*) and reverse δ^18^O showed annual and 5-year variations (fig. S18). A linear model was developed using 5-year mean Alpine scPDSI from 1901 to 2000 CE, with 1850 to 1900 CE used for verification. Deming linear regression (*67*) was applied to model the δ^18^O-scPDSI relationship (RStudio, Deming package). Our tree-ring δ^18^O chronology exhibits high hydroclimate sensitivity, consistent with several European (*2*, *24*, *25*, *68*) and global (*14*, *69*) proxy studies using tree-ring δ^18^O.

A monthly correlation analysis between tree-ring δ^18^O and temperature, precipitation, and scPDSI is shown in fig. S19 for the time window 1901–2000 CE. A monthly correlation analysis has been also performed between the tree-ring δ^18^O and the δ^18^O of the Global Network of Isotopes in Precipitation (GNIP) isotope precipitation measurements at Grimsel station without finding any significant correlation, neither in winter nor in summer months. The absence of significant correlation between tree-ring δ^18^O and precipitation δ^18^O indicates that evaporative processes, rather than precipitation isotopic composition, dominate the tree-ring oxygen isotope temporal variation (*70*) .

### Uncertainty evaluation

In our climate reconstruction, we identified and quantified four types of uncertainty: analytical, biological, calibration, and geographical. Analytical uncertainty, reflecting the precision of our measurement instruments, is denoted by a reported measurement precision of ±0.3‰ (*58*). Biological uncertainty, indicated by the SD of the annual δ^18^O signal, encapsulates the inherent variability in biological systems. Calibration uncertainty is expressed as the root mean square error in the linear relationship between δ^18^O values and Alpine scPDSI (*23*), highlighting the challenges in accurate calibration of our proxy data. Geographical uncertainty, influenced by the number and distribution of sampling regions, assesses the impact of localized or microclimatic effects on our chronology. This uncertainty is greater when fewer regions are sampled or when the regions are geographically close. As we consider all the errors independent, we combine them as the square root of the sum of the squares of the four individual errors to provide a clear overview of these uncertainties (fig. S20). Figure S20B illustrates the four types of errors, while fig. S20A presents the total error, calculated as the square root of the sum of the squares of these individual errors.

After confirming the independence of our values from the geophysical variables (latitude, longitude, and elevation), we proceeded to assess the significance of the Holocene JJA scPDSI trend. This was achieved by fitting a linear regression model to the data. To account for reconstruction error, we conducted a Monte Carlo simulation with 10,000 iterations, during which the model was fitted to JJA scPDSI reconstructions, incorporating uncertainty by randomly adding the error distribution. The inferred trend is significantly different from 0 (*P* ≤ 0.001) with a mean trend value of −0.226 JJA scPDSI/1000 years.

### Climate comparison

To validate and contextualize the JJA scPDSI, a correlation analysis was conducted with European hydroclimatic and temperature data from the past 500 years (table S4). This analysis involved the use of a 50-year rolling mean filter to assess the relationships between the JJA scPDSI and other relevant reconstructions. The results of the analysis revealed a strong positive correlation between the JJA scPDSI and two reference drought proxies: the JJA scPDSI reconstruction of Old World Drought Atlas (OWDA) (*71*) based on TRW (*r* = 0.53) and the JJA scPDSI reconstruction from central Europe based on TRSI (*2*) (*r* = 0.49). In addition, a negative correlation was observed between the reconstructed JJA scPDSI and temperature reconstructions (*46*, *72*, *73*). These results support the climate sensitivity analysis carried out for the 1901–2000 period. An analysis of the past 2000 years reveals a significant positive correlation of our data with the central European JJA scPDSI from a TRSI reconstruction (*2*) and a significant negative correlation with temperature reconstruction based on TRW (*74*).

We further compare our reconstruction with other climate reconstructions from Europe that span the past 9000 years. We collected all precipitation and temperature data from the Alps for the Holocene used for a global hydroclimate reconstruction (*75*) and found that all reconstructions have a large degree of uncertainty (fig. S21). However, a good agreement has been found with the evapotranspiration anomaly reconstructed from pollen records for the Alps (*76*) (fig. S9). Our hydroclimate reconstruction has a negative Holocene-long trend similar to that observed in other regional climate reconstructions based on water stable isotopes (δ^18^O and δ^2^H) (fig. S20), whereas less agreement is found with the speleothem precipitation reconstructions (fig. S22).

The traditional calibration-verification method, focusing on short-term correlations (figs. S3 and S5 and table S3), provides the highest correlation with scPDSI and a positive correlation with temperature. However, the positive correlation between temperature and tree-ring δ^18^O seems to contradict the final Holocene climate comparison. In this comparison, our reconstructed TRSI-based JJA scPDSI shows a similar long-term trend to temperature reconstructions based on water isotopes from ice caps and speleothems (fig. S4, S23). The final scPDSI values have an inverse relationship compared to the original tree-ring δ^18^O, as the original correlation is negative.

The long-term increase in tree-ring cellulose δ^18^O in this area can be explained primarily by long-term drying. There are two main factors controlling tree-ring cellulose δ^18^O: precipitation δ^18^O and leaf water δ^18^O enrichment due to transpiration. Given that precipitation δ^18^O decreased over the Holocene in this region, as illustrated by fluid inclusion water isotope measurements in stalagmites (*20*), long-term drying must be the cause of the long-term increase in tree-ring cellulose δ^18^O through leaf water δ^18^O enrichment. This can be only explained by a mechanical model of isotope fractionation, as illustrated in Fig. 2, regardless of the timescale of climate variations. Furthermore, the comparison between our tree-ring δ^18^O chronology and the isotopic signal of precipitation extracted from the isotope chronology of fluid inclusions from Swiss speleothems (*20*) confirms that the isotopic trends in the two records are inversely related. This is evident from the opposing values observed in the tree-ring δ^18^O data (fig. S23).

Modeled precipitation rates were analyzed in a specific region between 44° and 50°N and 5° and 20°E. Total precipitation, convective precipitation, and large-scale precipitation data were obtained from the TraCE-21K-II simulation (Transient Climate Evolution of the past 21,000 years), a fully forced climate model (*77*). The precipitation data were extracted from https://trace-21k.nelson.wisc.edu/portal.html and averaged spatially to derive mean precipitation rates for the area of interest. These measurements were aligned with our TRSI scPDSI data and aggregated to the same time resolutions for correlation analysis (fig. S8). The correlation between total precipitation rate and scPDSI is 0.29 (*P* < 0.05), the correlation between scPDSI and convective precipitation rate is 0.31 (*P* < 0.05), and the correlation between scPDSI and large-scale precipitation rate is −0.14 (*P* < 0.05).

## Supplementary Materials

### This PDF file includes:

Supplementary Text Figs. S1 to S23 Tables S1 to S4 References
