# Supplementary material for: Tree-ring stable isotopes from the European Alps reveal long-term summer drying over the Holocene
Source: Sci Adv. 2025 Apr 4;11(14):eadr4161. doi: 10.1126/sciadv.adr4161 (PMC11970467; doi:10.1126/sciadv.adr4161)
Supplement: Supplementary file 1 — Supplementary Text Figs. S1 to S23 Tables S1 to S4 References [file sciadv.adr4161_sm.pdf]

Science Advances
AAAS

Supplementary Materials for

Tree-ring stable isotopes from the European Alps reveal long-term summer drying over the Holocene

Tito Arosio *et al.*

Corresponding author: Tito Arosio, tito.arosio87@gmail.com

Sci. Adv. **11**, eadr4161 (2025)
DOI: 10.1126/sciadv.adr4161

This PDF file includes:

Supplementary Text
Figs. S1 to S23
Tables S1 to S4
References

Hydroclimate implication

Our TRSI record provides evidence of relatively stable hydroclimate conditions around 7600 and 6900 BP that further correspond temporally with the emergence and consolidation of the first agricultural communities in central Europe, known as Linear Pottery Culture (LBK) (*80*) (Fig. S11). A relatively dry period around 6700 BP aligns with a phase of social fragmentation within these early agricultural communities, although economic foundations and settlement systems remain unchanged (*81*). The earliest archaeological evidence for a high-elevation Alpine crossing over the Schnidejochpass in the western Swiss Alps level falls into the same dry period at 6700 BP (Fig. S11) (*82*), which also marks the onset of the so-called Chamblandes-type graves in the western Alps (*83*). Their presence in the inner-alpine Rhone and Aosta valleys, as well as around Lake Geneva, provide evidence of intensive settlement development and transalpine exchange. A prolonged wet period from around 5300–4700 BP in the western Alps is characterized by economic innovations, new forms of cult and burial, as well as a high degree of mobility and change, including the beginning of megalithic centers across the main Alpine ridge in the Rhone and Aosta valleys (*84*). Economic development is indicated by first finds of wheels, ploughs and yokes from lakeshore settlements, and an increase in arable land, but also in livestock farming, especially pig farming (*85*). The transition between a wet phase at 5000 BP and a dry and cold (*38*) phase at 4600 BP overlaps with a major genetic reshuffling of European populations, probably linked to the immigration of Yamnaya people from the Pontic steppe (*86*). Another period of drought around 4200 BP, often described as the 4.2ka event (*87*), not only corresponds with the decline of the Bell Beaker phenomena (*88*), but also with a major gap in lake dwellings around the Alpine arc (Fig. S11). The Celtic migration around 2300 BP, the fall of the Roman Empire, and large-scale human

movements between the 4th and 6th centuries AD, as well as a series of societal changes associated with the LALIA and LIA, all fall into generally drier periods.

Supplementary figures S1–23

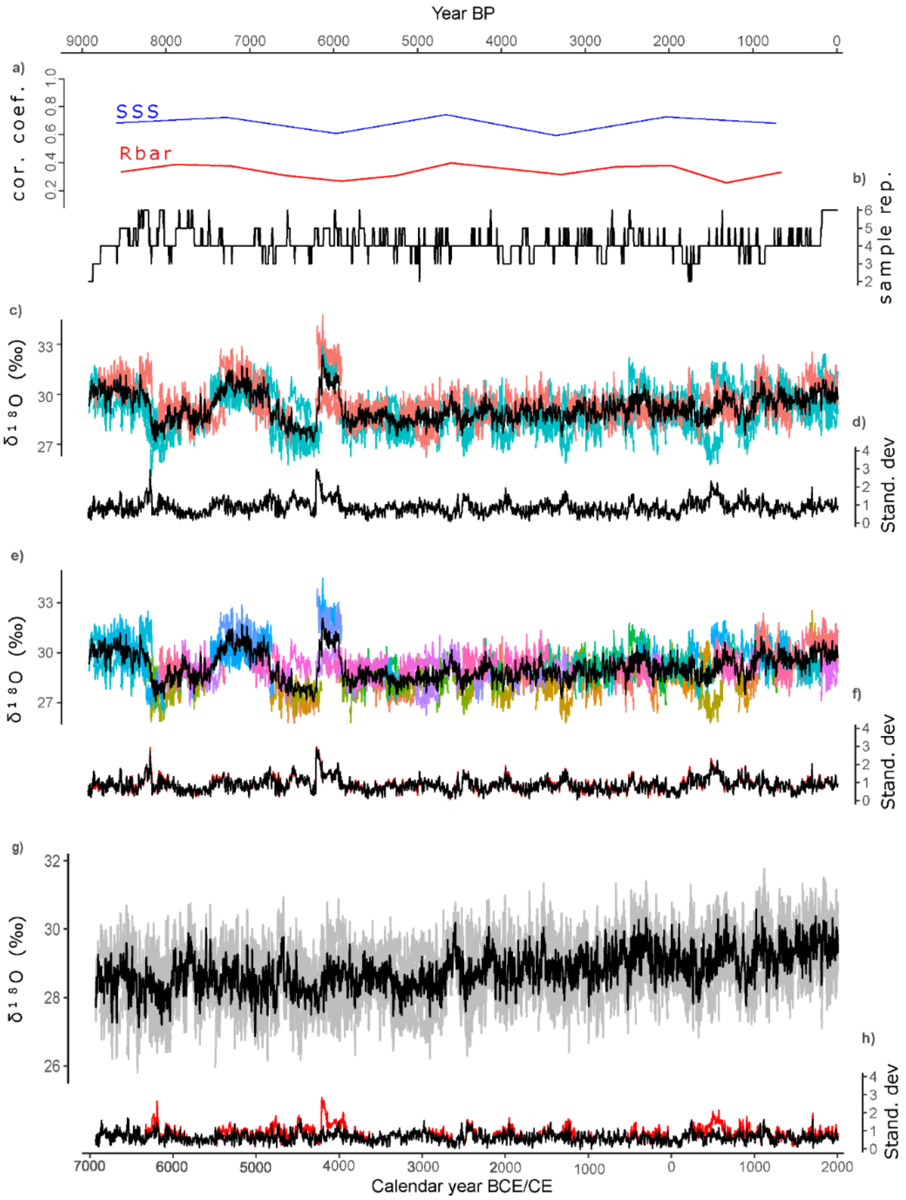

Figure S1. Corrections for the development of the chronology.(**a**) Running SSS (blue) and Rbar (black) of the $\delta^{18}O$ dataset, calculated over a 250-year window. (**b**) The sample depth of the $\delta^{18}O$ chronology. (**c**) Visualization of all 192 time-series colored by the species: larch samples in orange and cembran pine samples in cyan, with their mean values in black. (**d**) Standard deviation of $\delta^{18}O$ as a function of time. (**e**) Visualization of all-time series data after the age-trend correction, each color represents a time series; and black is for the mean value. (**f**) Standard deviation of the age-trend corrected chronology in black (panel d) and the standard deviation of the uncorrected chronology in red (panel c). (**g**) Visualization of all-time series after the corrections (gray) and the mean value in black. (**h**) Standard deviation of the age-trend and offset corrected chronology in black and the standard deviation of the age-trend corrected chronology in red (panel e).

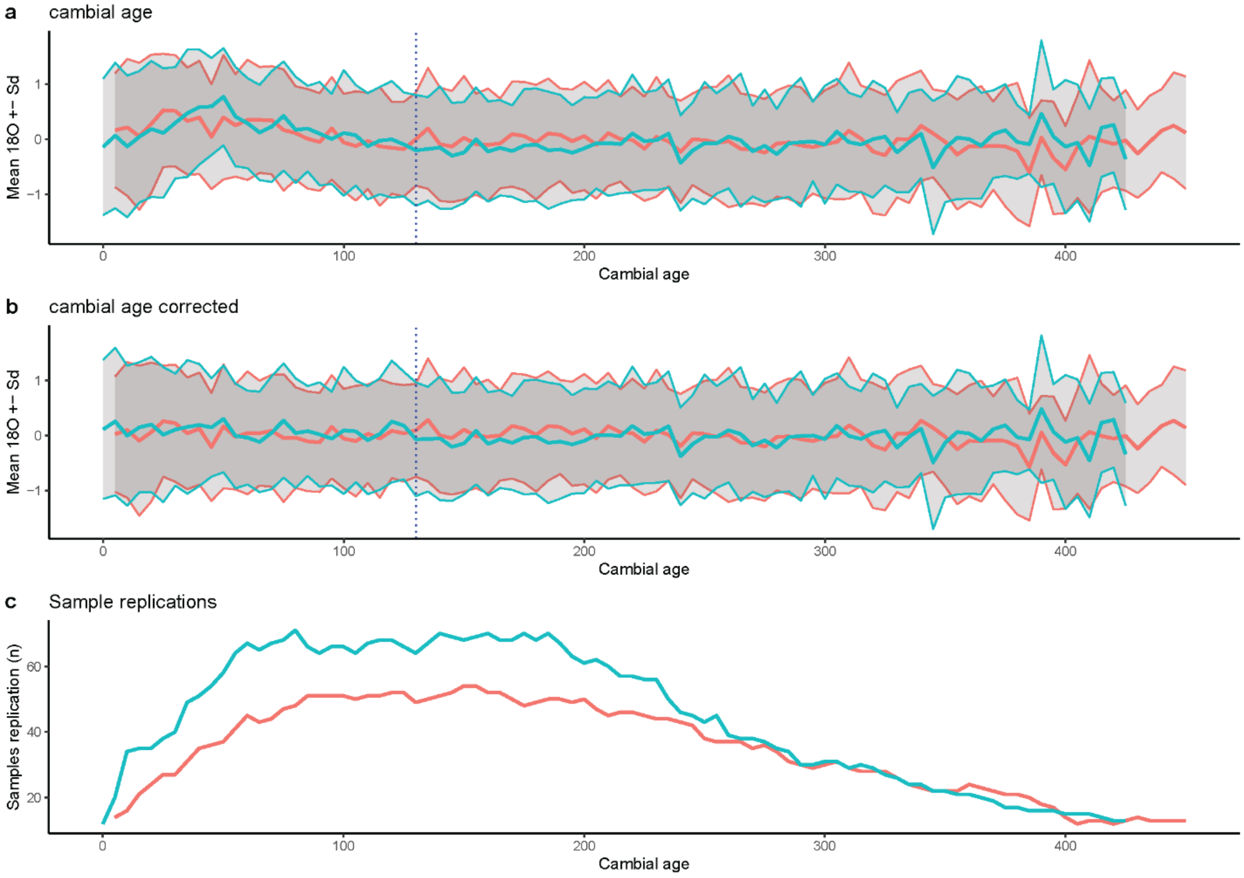

Figure S2. **Cambial age-trend correction.**
(**a**) Normalized raw and mean values of the $\delta^{18}O$ individual series aligned by cambial age with corresponding ±1 standard deviation (gray area) of larch (red) and cembran pine (cyan). (**b**) Normalized mean value with corresponding ±1 standard deviation (gray area) of larch (LADE) and cembran pine (PICE) after the age-trend correction. (**c**) Number of samples per cambial age with a threshold of 10 that we accepted for the analysis. The dotted line represents the cambial age of 130 years.

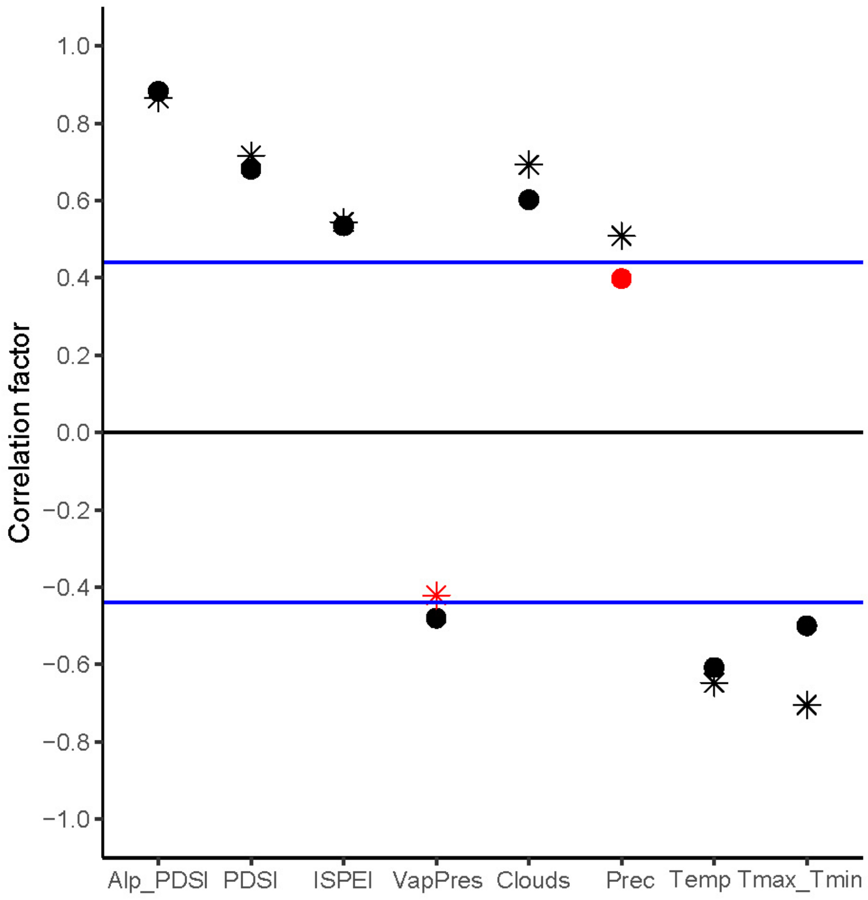

Figure S3. Correlation factors of the δ^{18}O TRSI (inverse) for the 1901–2000 CE period: *Alps scPDSI*: Alpine JJA scPDSI averaged for all the four alpine regions (*23*). *PDSI:* CRU JJA scPDSI; *ISPEI*: CRU JJA SPEI; *VapPres*: CRU JJA Vapour Pressure range; *Clouds*: CRU JJA cloud cover; *Prec*: CRU JJA precipitation; *Temp*: CRU JJA temperature anomaly; *Tmax_Tmin (Dtr)*: CRU JJA daily temperature range. All climate variables are averaged over 46–47° N and 7.55–12.25° E. The dots indicate the correlation of the raw data, and the asterisk indicates the correlation of the first-time difference. The red color is for non-significant correlation (p >0.05), black color represents a significant correlation (p ≤0.05), the blue lines indicate the critical value of r =0.05.

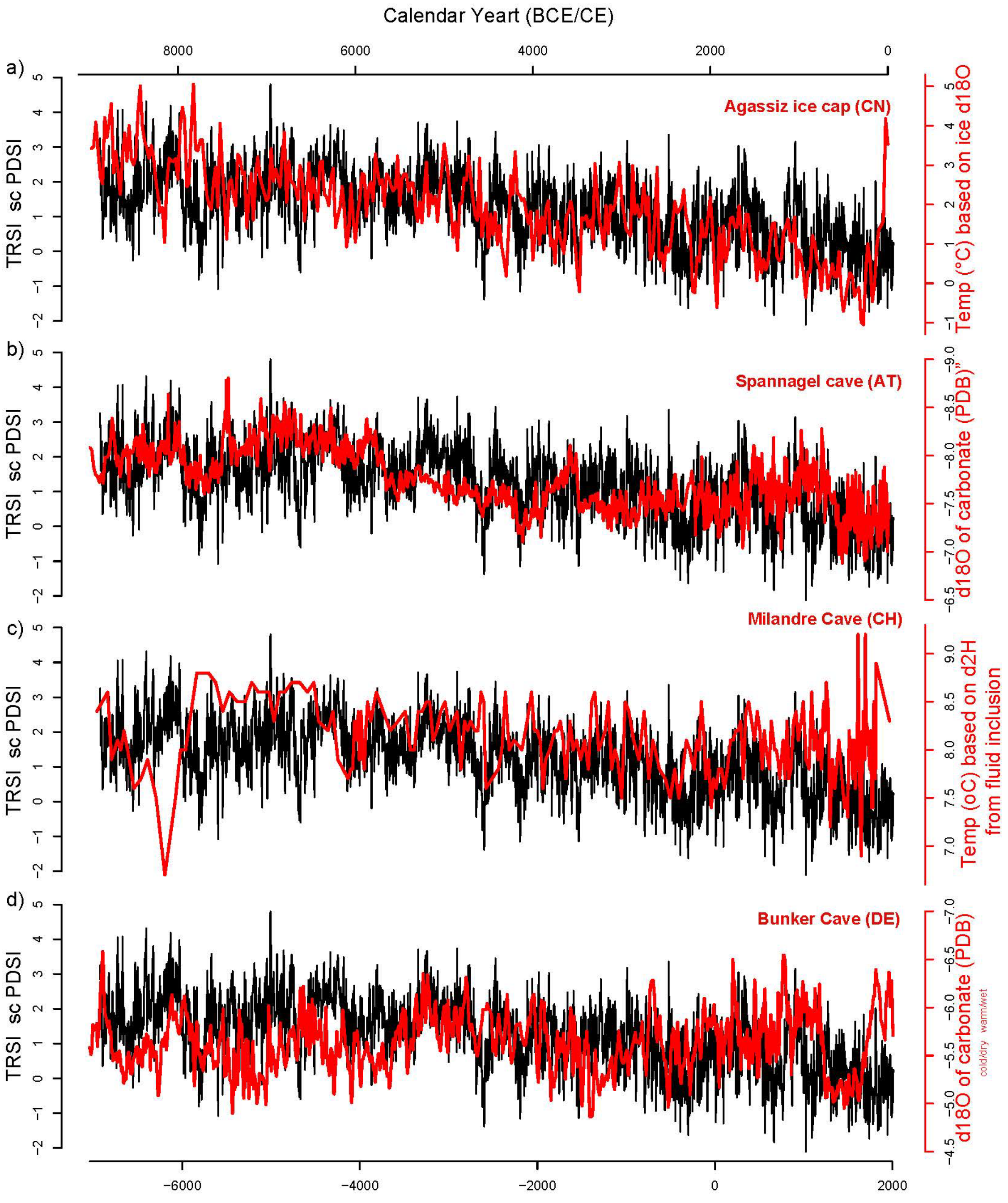

Figure S4. Multi-millennial scPDSI reconstruction from this study (in black) compared with previously published water isotope-based temperature records from ice cap in Canada and caves across Europe spanning the last 8,000 years (in red): (**a**) Agassiz ice cap record from Ellesmere Island, Canada, based on $\delta^{18}O$ (*21*), (**b**) Spannagel Cave record from Austria (*38*), (**c**) Milandre Cave record from Switzerland (*20*), (**d**) Bunker Cave record from Germany (*38*). In our reconstruction, negative scPDSI values indicate higher $\delta^{18}O$ values, while positive values indicate lower $\delta^{18}O$ values.

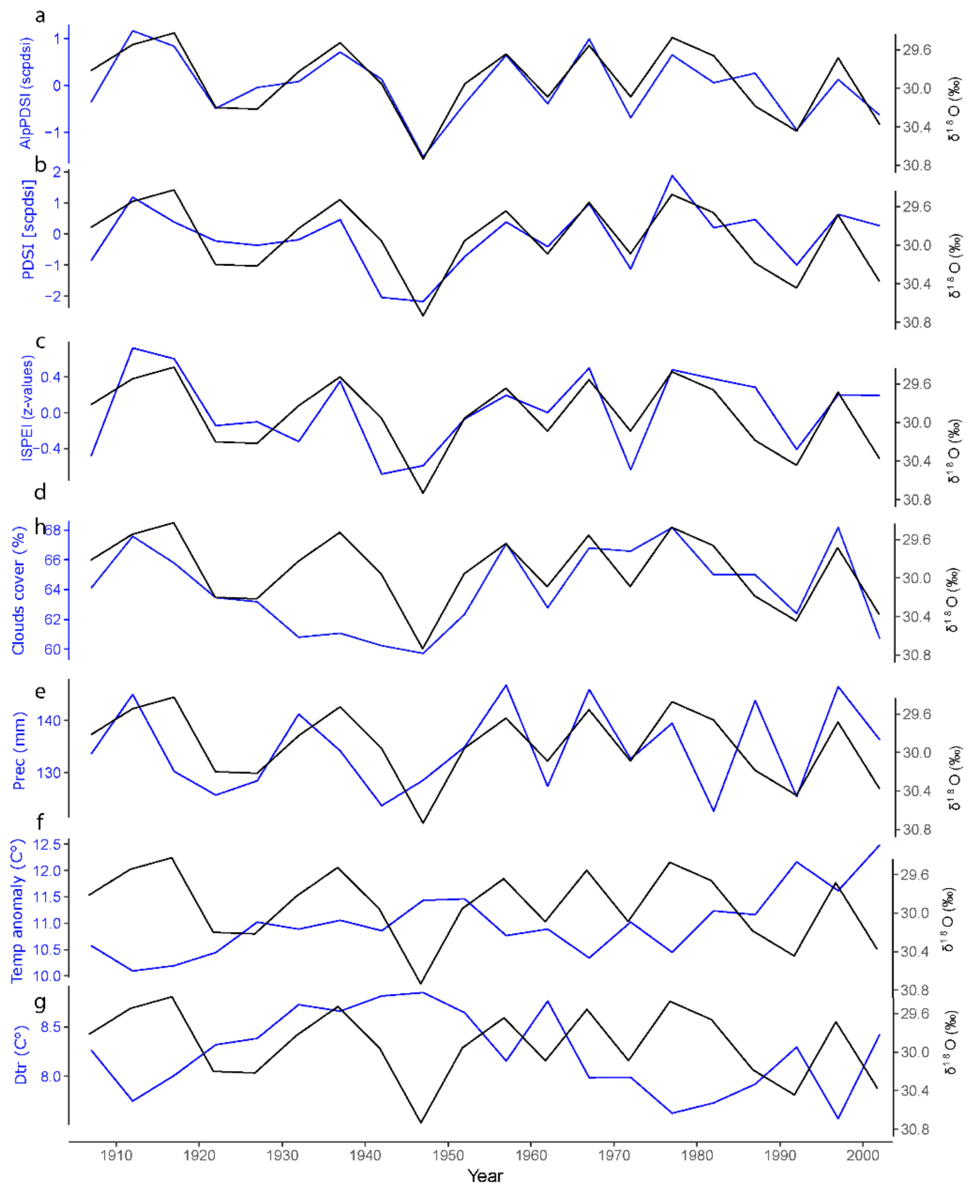

Figure S5. Comparison between climate variables (blue lines) and the inverse δ^{18}O TRSI (black lines). Climate variable interpolated with a five-year resolution, from the top: (**a**) *AlpPDSI*: Alpine JJA scPDSI measured and averaged for all the four alpine regions (*23*); (**b**) *PDSI:* CRU JJA scPDSI; (**c**) *ISPEI*: CRU JJA SPEI; (**d**) *Clouds cover*: CRU JJA cloud cover; (**e**) *Prec*: CRU JJA precipitation; (**f**) *Temp anomaly*: CRU JJA temperature anomaly; (**g**) *Dtr*: CRU JJA daily temperature range; (**h**) *Vapour Pressure*: CRU JJA Vapour Pressure range. All climate variables are averaged over 46–47° N and 7.55–12.25° E.

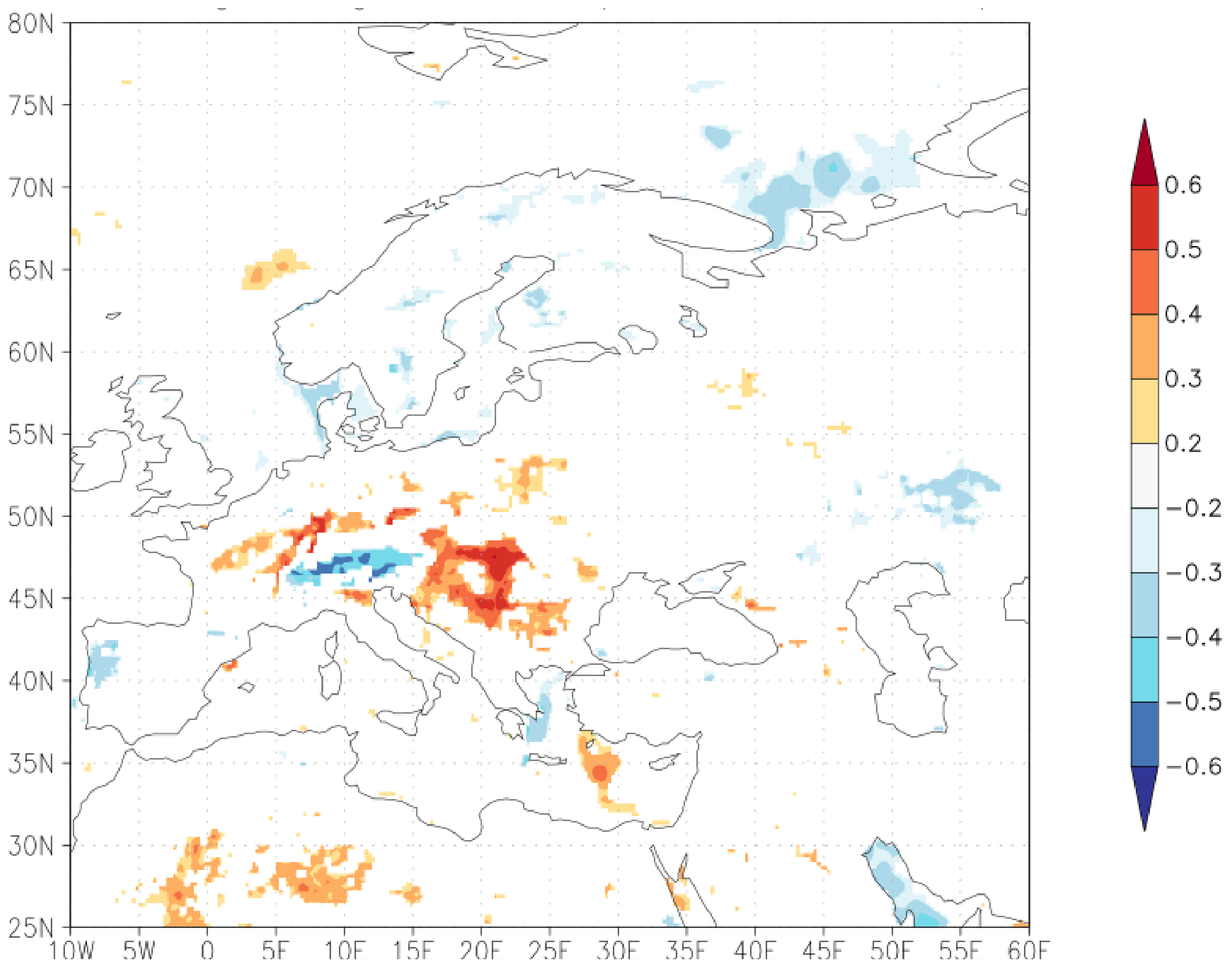

Figure S6. Spatial correlation analysis between potential evapotranspiration and the Alpine JJA scPDSI. High-resolution spatial correlation coefficients (color scale) between the annually resolved Alpine JJA scPDSI target data and the 0.5°×0.5° gridded CRU European-wide July-August potential evapotranspiration over the common period 1950–2000 CE.

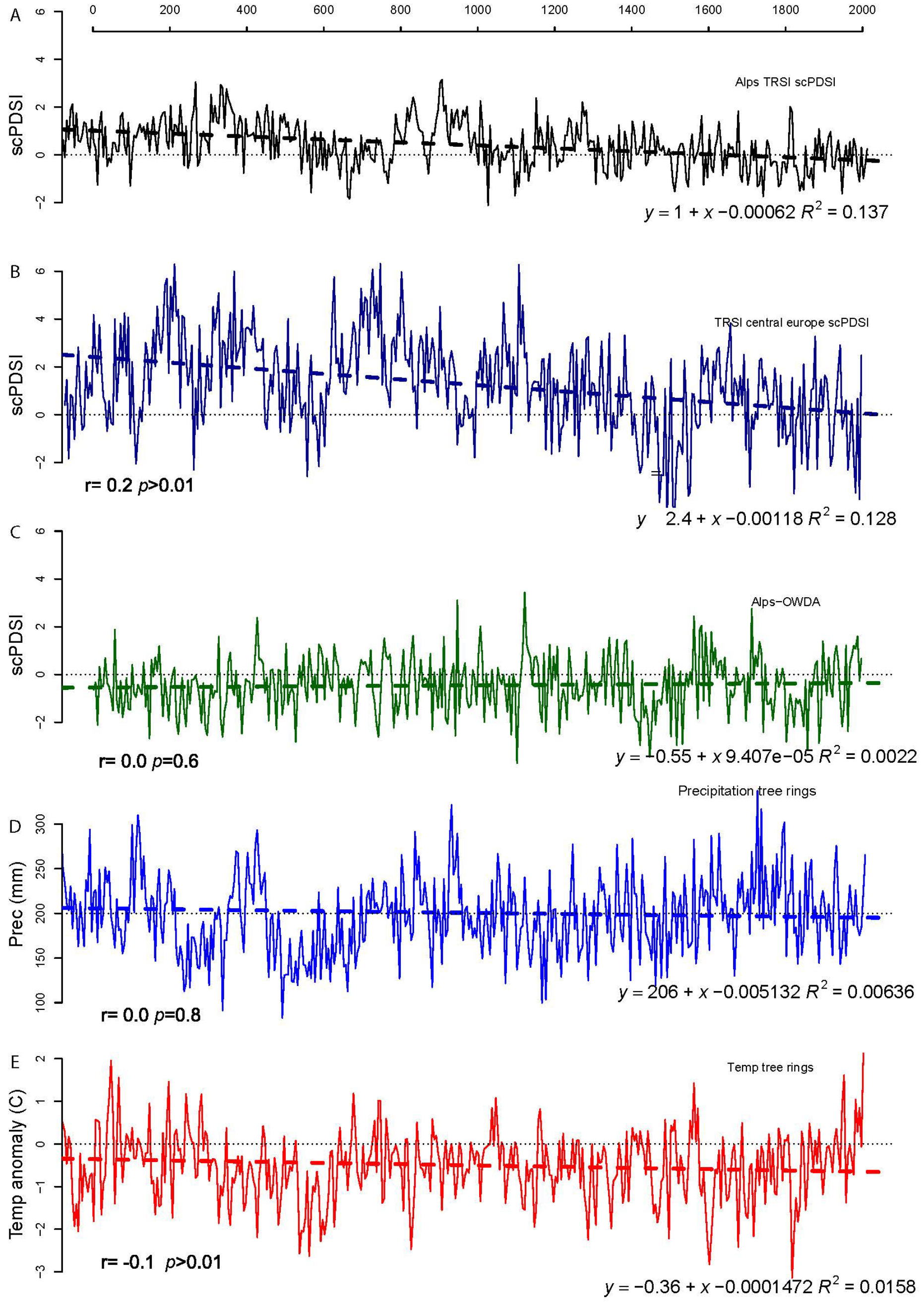

Figure S7. Three tree-ring-based scPDSI reconstructions of the past 2,000 years for Europe. (**A**) Present study with a five-year resolution. (**B**) scPDSI reconstruction based on $\delta^{18}O$ and $\delta^{13}C$ for central Europe, interpolated to a five-year resolution (*2*). (**C**) OWDA scPDSI reconstruction for the 46–47° N and 7.55–12.25° E based on TRW interpolated to a five-year resolution (*71*). **(D)** Precipitation reconstruction for central Europe based on TRW, interpolated to a five-year resolution (32). (**E**) Temperature reconstruction for central Europe based on TRW, interpolated to a five-year resolution (32). For each reconstruction, a linear interpolation, a formula, a correlation factor, and a p-value are shown for the correlation with the present study.

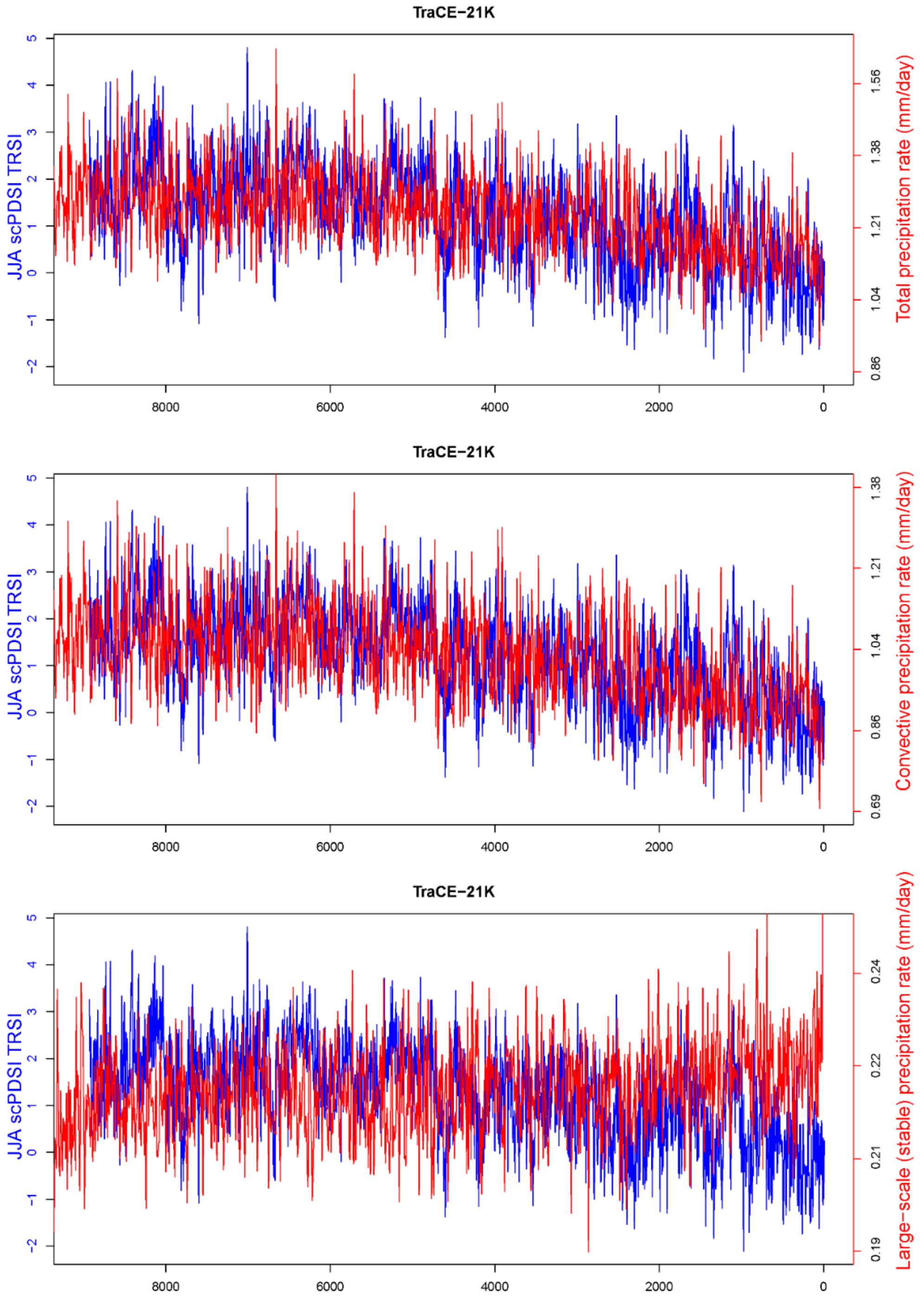

Figure S8. Multi-millennial tree-ring (TR) δ^{18}O from this study (in blue) compared with time-series analysis from the TraCE-21K precipitation fully-forced simulation dataset (https://trace-21k.nelson.wisc.edu/portal.html).The TraCE-21K are the decadal mean values for the grid 46–47° N and 7.55–12.25° E (in red), the top panel showing total precipitation, the middle panel showing convective precipitation, and the bottom panel showing large-scale (stable) precipitation.

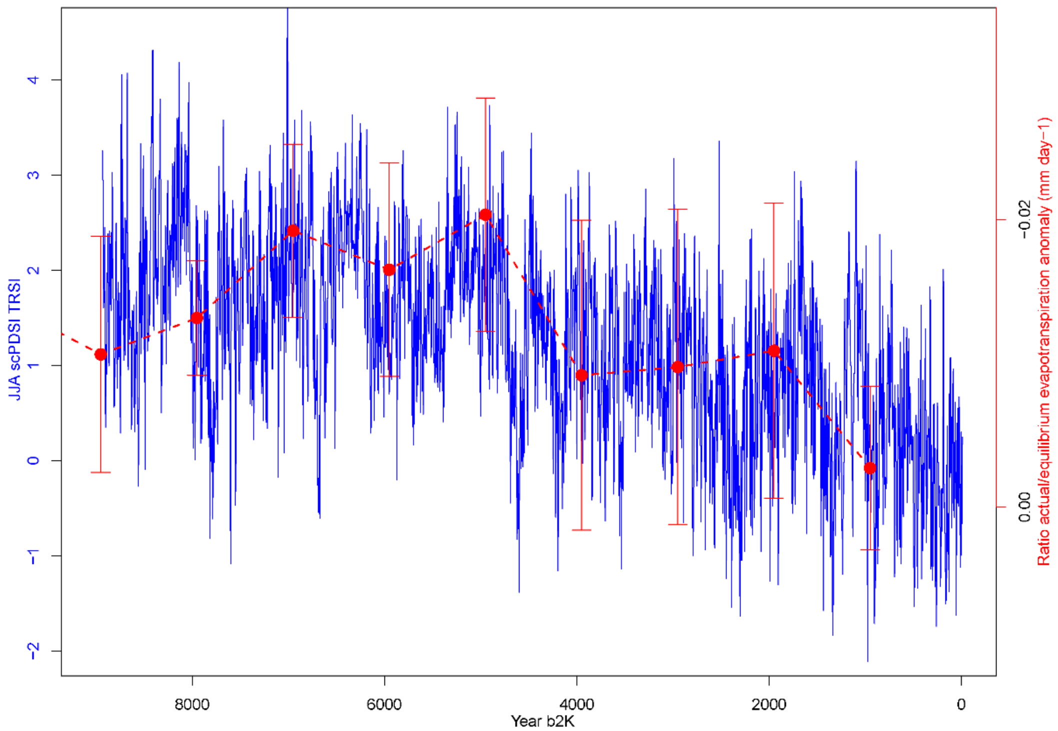

Figure S9. Comparison of the JJA scPDSI (in blue) and the reconstructed Holocene mean annual ratio equilibrium evapotranspiration anomaly (mm/day) (in red) (*76*) for the grid between 46° 03' – 47°05' N and 7.55° – 12.25° E.

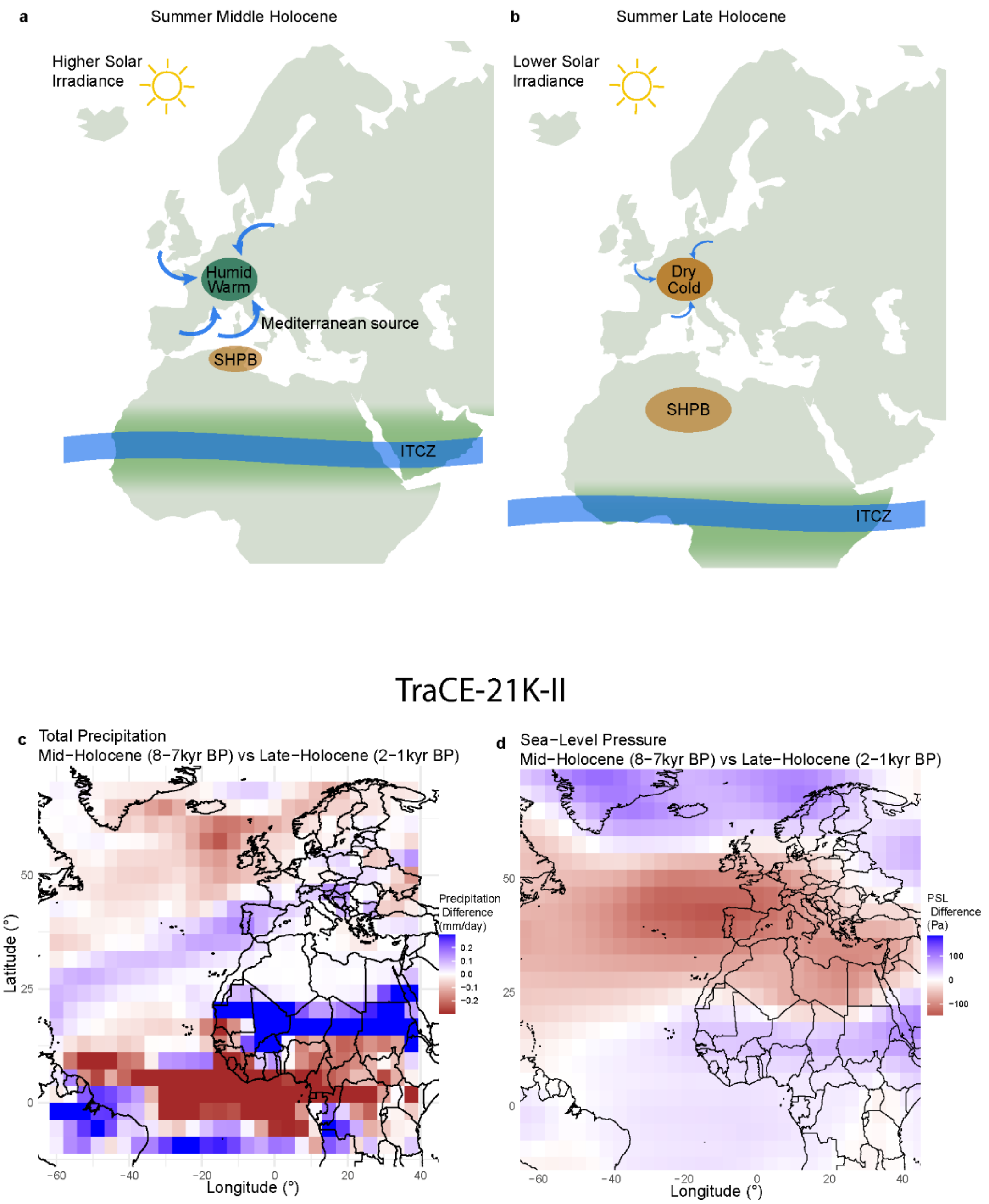

Figure S10. Schematic representation of the summer atmospheric conditions during the mid-Holocene (a) and the late Holocene (b) as hypothesised in this study and TRACE-21k-II simulations. The ITCZ is the Inter Tropical Convergence Zone, SHPB is a subtropical high-pressure belt. Differences in annual precipitation (c) and annual pressure at sea level (PSL) (d) from TraCE-21k II simulations between the mid-Holocene (8–7 kyr BP) and the late Holocene (2–1 kyr BP). Red shades indicate drier conditions (c) or higher pressure (d) in the mid-Holocene compared to the late Holocene. Blue shades indicate wetter conditions (c) or lower pressure (d) in the mid-Holocene.

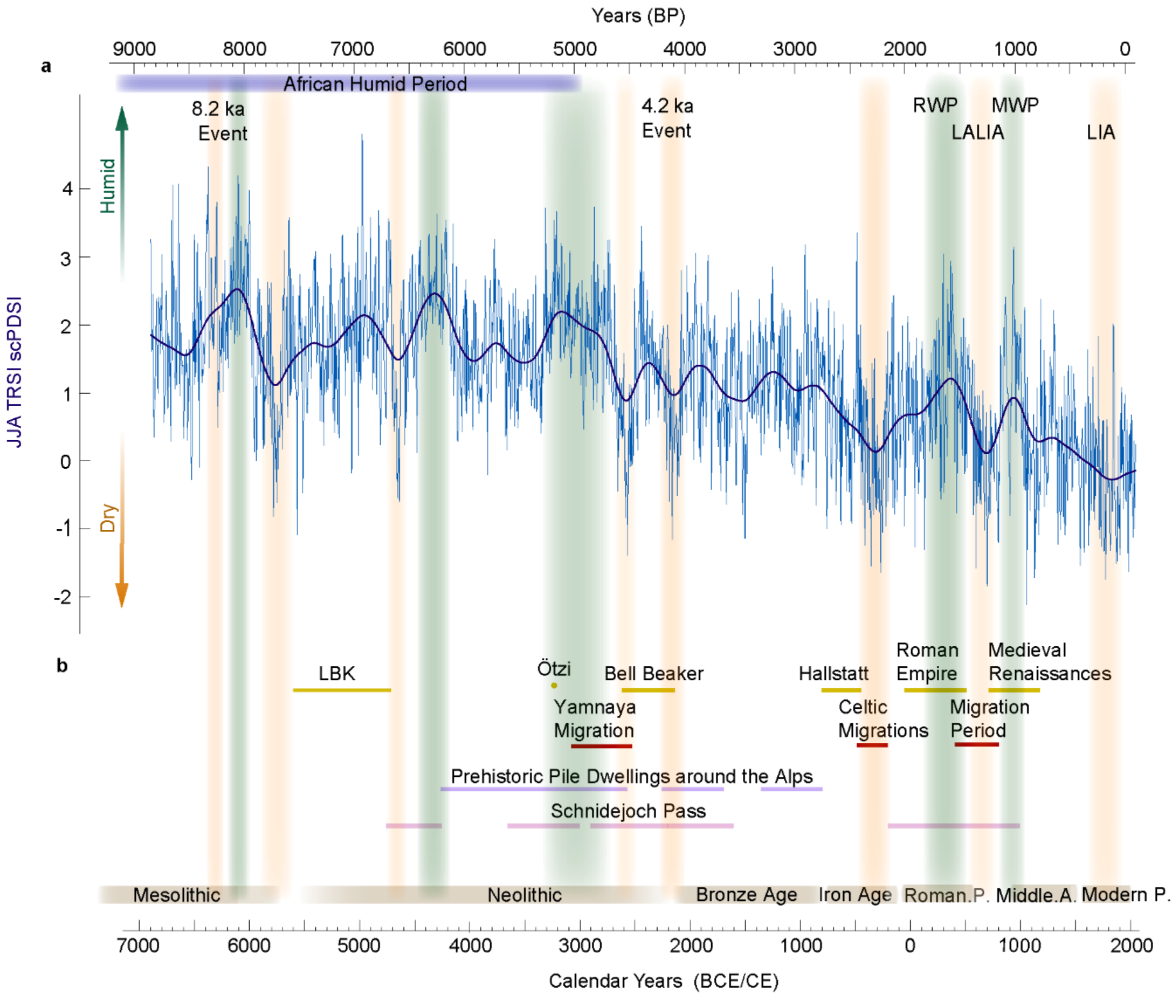

Figure S11. **Causes and consequences of Alpine summer hydroclimate changes over the Holocene**. On the top, prominent climatological phases are shown that are characteristic for the past 9,000 years in central Europe and the Alpine arc, including the African Humid Period, the 8.2 and 4.2 ka Events, the Roman Warm Period (RWP), the Medieval Warm Period (MWP), the Late Antique Little Ice Age (LALIA), and the Little Ice Age (LIA). (**a**) Our $\delta^{18}O$ TRSI-based JJA scPDSI reconstruction from the European Alps (dark blue) and its 500-year low-pass filter (smoothed line). (**b**) Horizontal bars refer to prominent archaeological phases that are characteristic for the past 9,000 years in central Europe and the Alpine arc, including periods of increased human population (brown), migrations (red), humans crossing the Schnidejoch Pass in the Swiss Alps (pink), pile dwelling cultures on the pre-alpine lakes (purple), and Linear Pottery Culture (LBK).

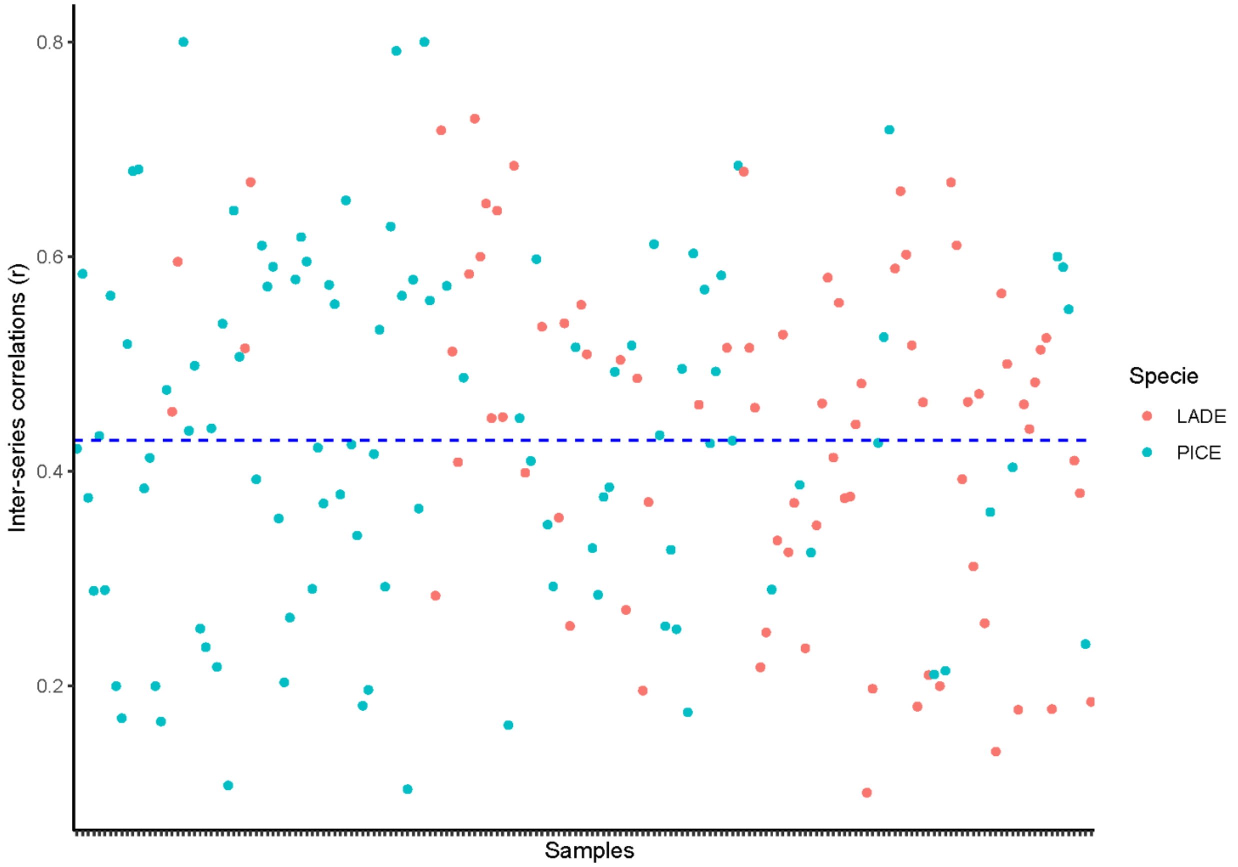

Figure S12. **Inter-series correlation for each sample.** Inter-series correlation (r) for each larch (red dots) and cembran pine (cyan dots) sample. The dotted blue line is the mean inter-series correlation.

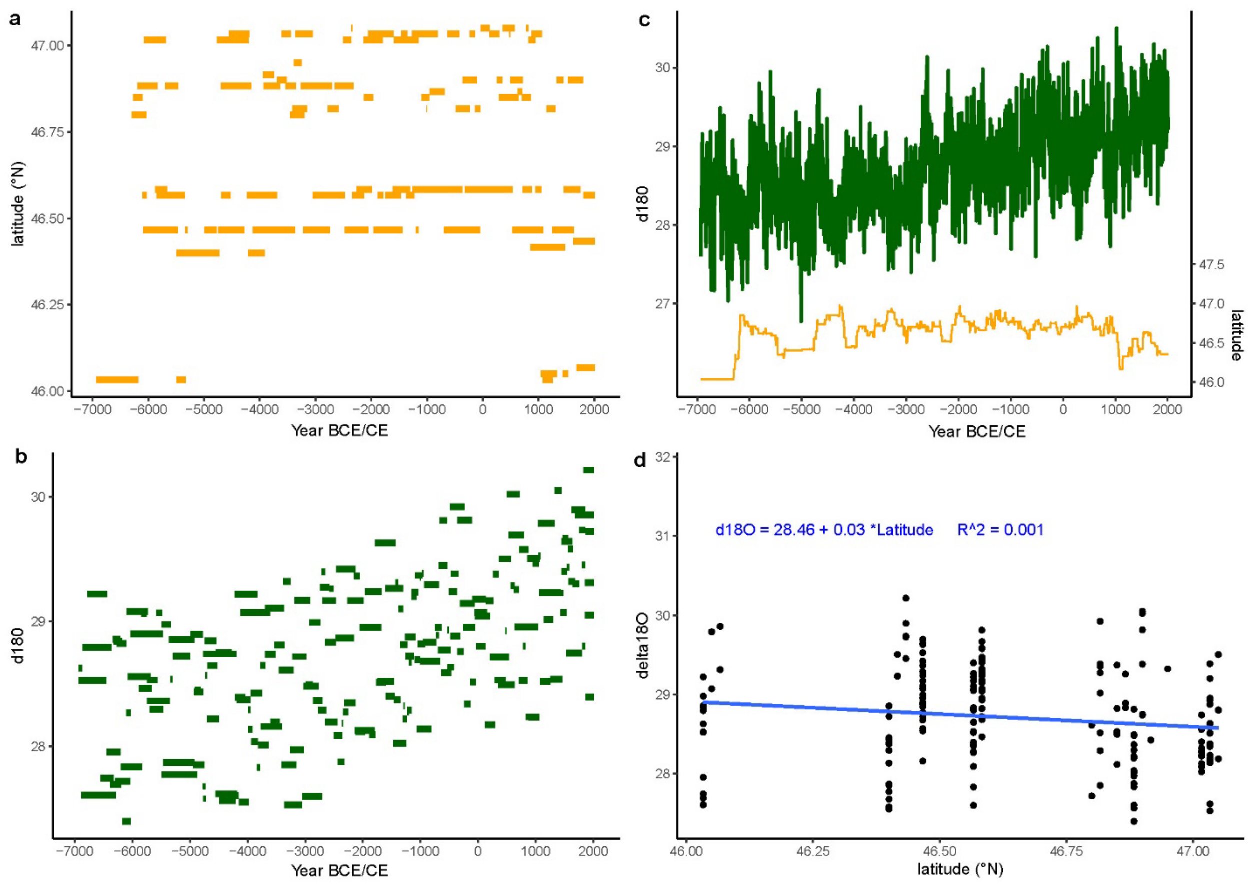

Figure S13. Relationship between δ^{18}O corrected series and samples' latitude over the past 9,000 years. (**a**) Sampled tree's latitude and a time-series length of each tree. (**b**) Series mean δ^{18}O and a time-series length of each tree. Each floating bar represents one δ^{18}O series and has a length indicating the length of the sample series. (**c**) Arithmetic means of the series' means (in green, as in (b)) and of the series' latitudes (in orange, as in (a)). (**d**) Scatterplot showing the overall relationship between the mean δ^{18}O values of each series and sampled tree's latitude.

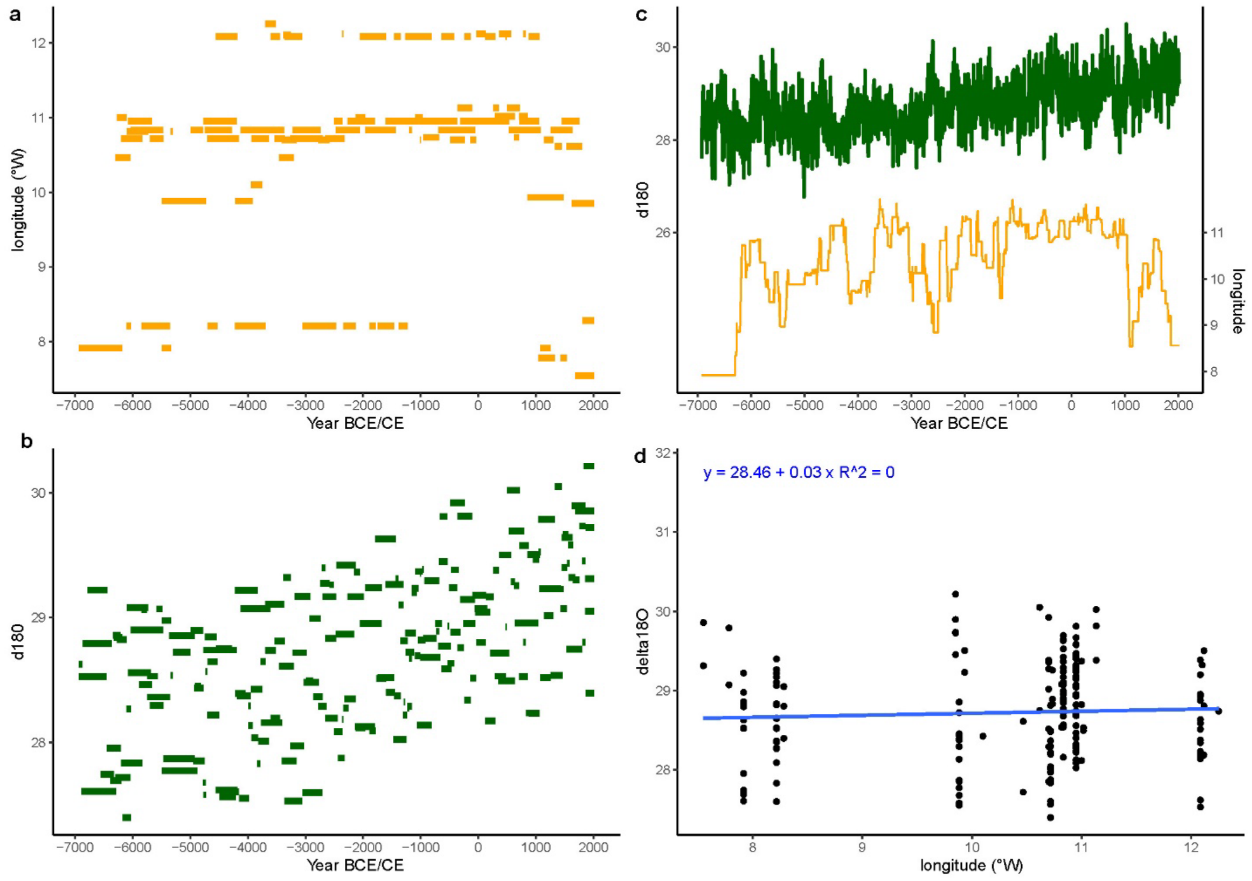

Figure S14. Relationship between δ^{18}O corrected series and samples' longitude over the past 9,000 years. (**a**) Sampled tree's longitude and a time-series length of each tree. (**b**) Series mean δ^{18}O and a time-series length of each tree. Each floating bar represents one δ^{18}O series and has a length indicating the length of the sample series. (**c**) Arithmetic means of the series' means (in green, as in (b)) and of the series' longitudes (in orange, as in (a)). (**d**) Scatterplot showing the overall relationship between the mean δ^{18}O values of each series and sampled tree's longitude.

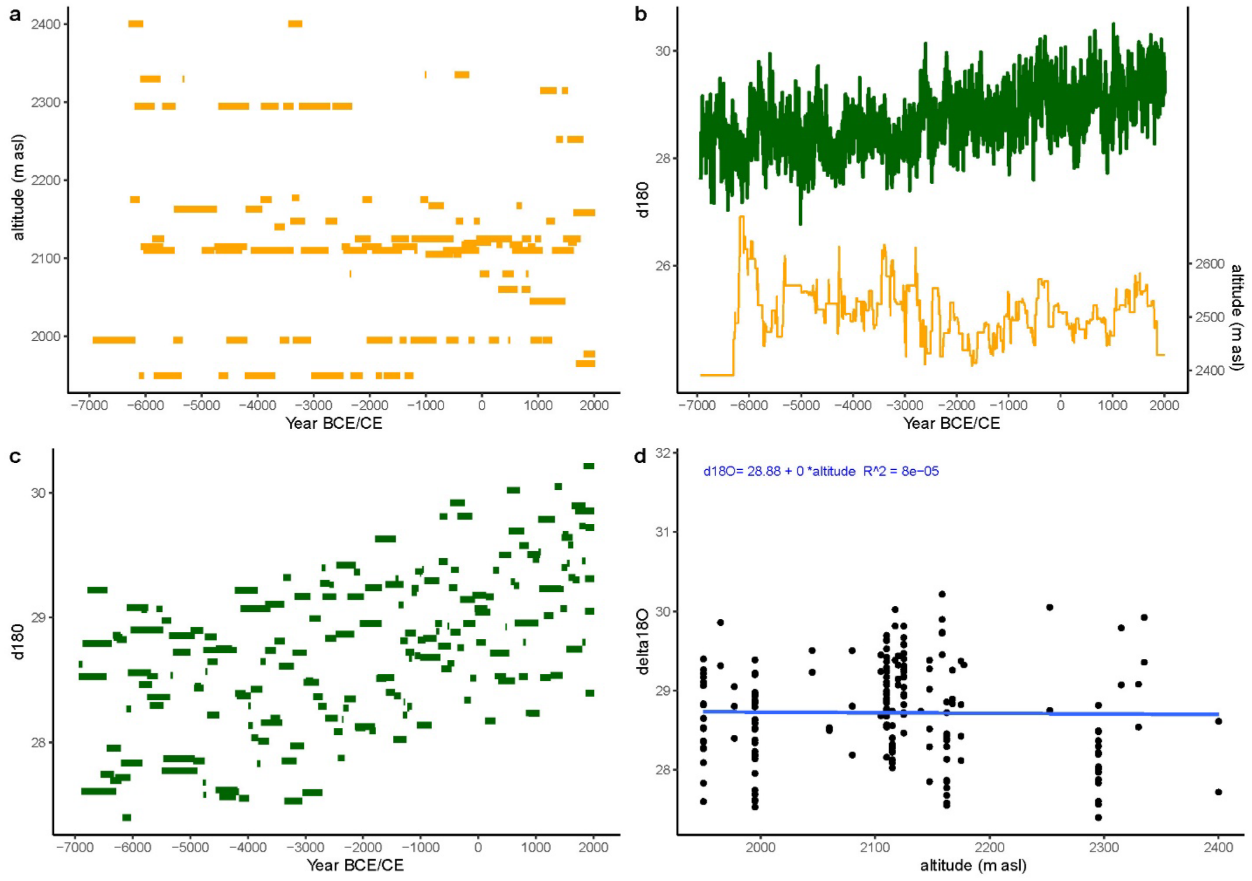

Figure S15. Relationship between $\delta^{18}O$ corrected series and samples' elevation over the past 9,000 years. (**a**) Sampled tree's elevation and a time-series length of each tree. (**b**) Series mean $\delta^{18}O$ and a time-series length of each tree. Each floating bar represents one $\delta^{18}O$ series and has a length indicating the length of the sample series. (**c**) Arithmetic means of the series' means (in green, as in (b)) and of the series' elevation (in orange, as in (a)). (**d**) Scatterplot showing the overall relationship between the mean $\delta^{18}O$ values of each series and sampled tree's elevation.

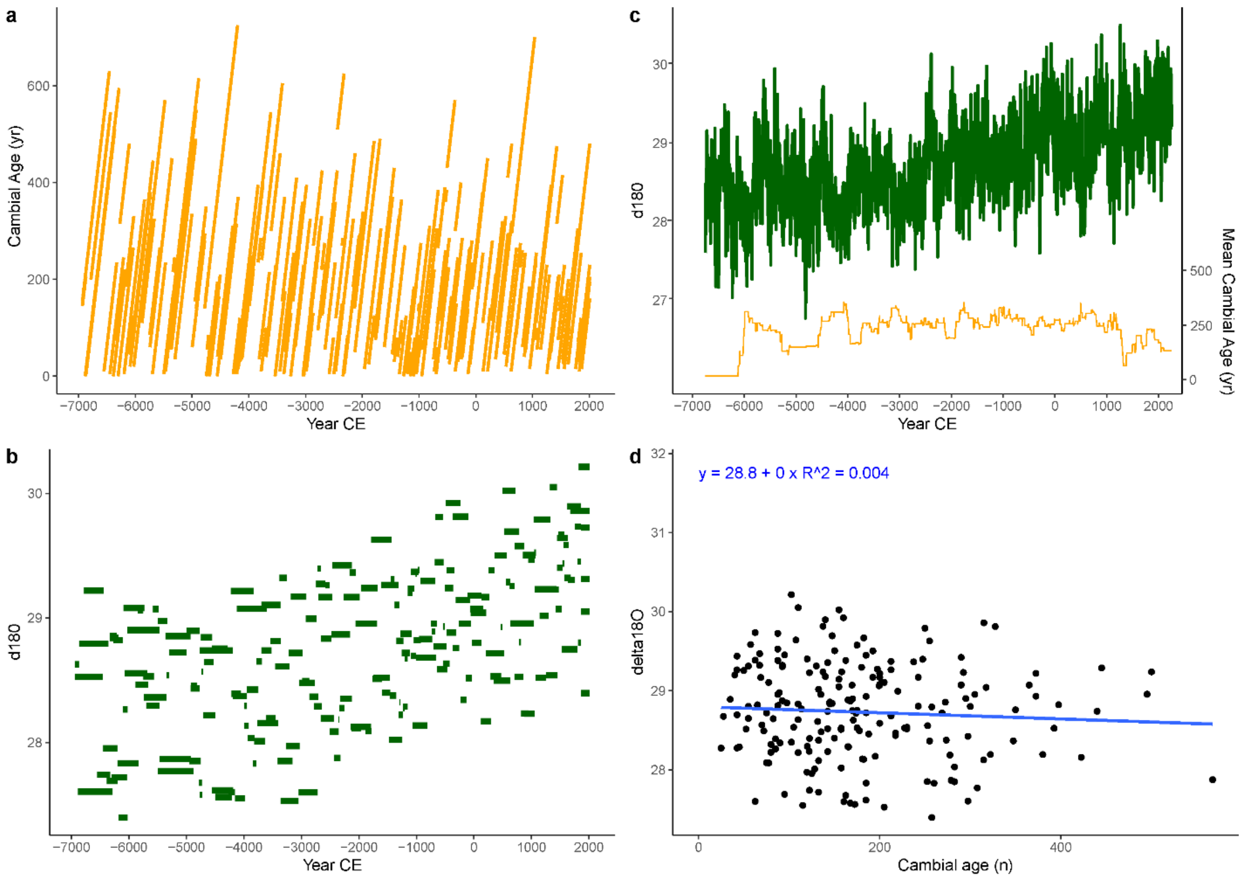

Figure S16. Relationship between δ^{18}O corrected series and cambial age over the past 9,000 years. (**a**) Temporal distribution of all trees and their cambial age. (**b**) Series' mean δ^{18}O and a time-series length of each tree. Each floating bar represents one δ^{18}O series and has a length indicating the length of the sample series. (**c**) Arithmetic means of the series' means (in green, as in (b)) and of the series' cambial age (in orange, as in (a)). (**d**) Scatterplot showing the overall relationship between the mean δ^{18}O values of each series and cambial age.

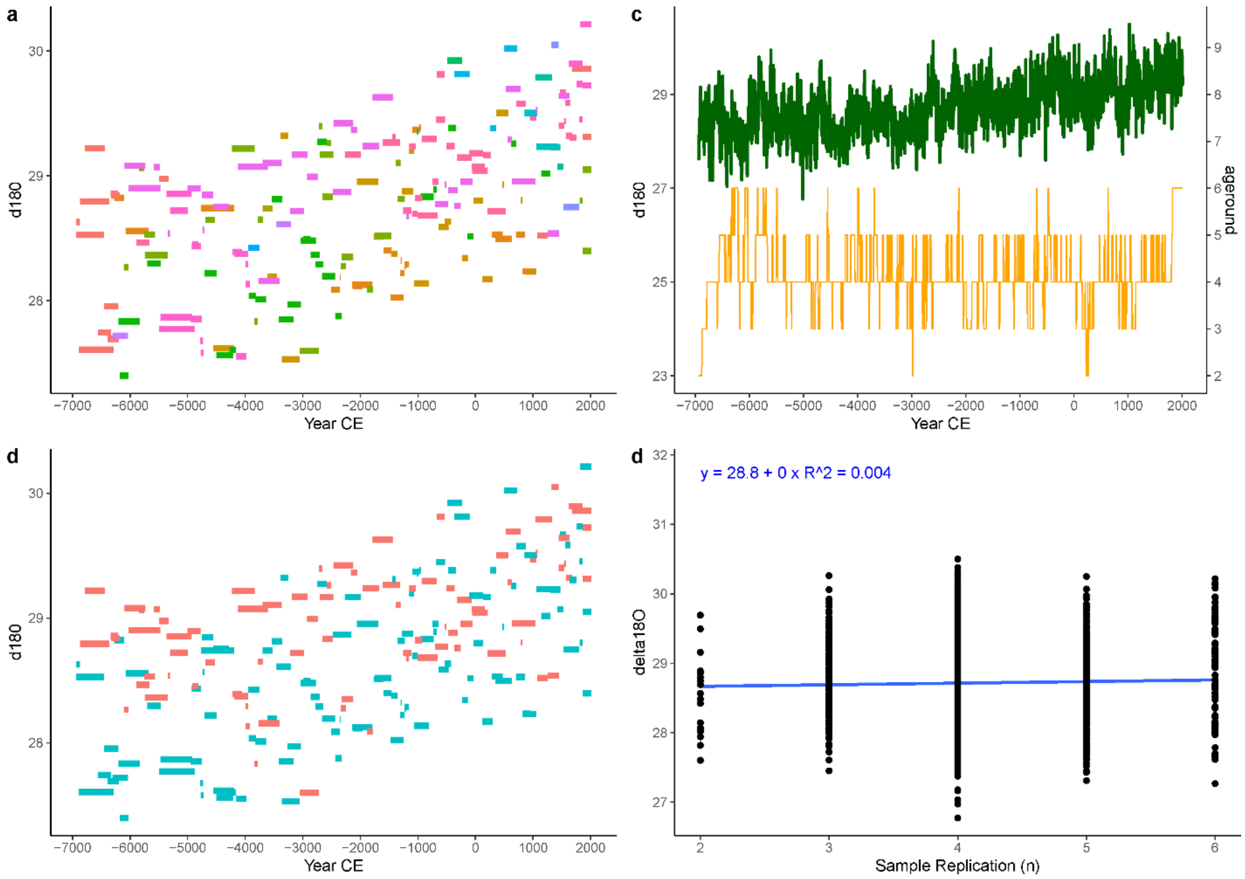

Figure S17. Relationship between δ^{18}O corrected series and sample replication over the past 9,000 years. (**a**) Series' mean δ^{18}O and a time-series length of each tree, the colours represent the 29 sampling sites. Each floating bar represents one δ^{18}O series and has a length indicating the length of the sample series. (**b**) Series' mean δ^{18}O and a time-series length of each tree, the colours represent the two species LADE (red) and PICE (cyan). Each floating bar represents one δ^{18}O series and has a length indicating the length of the sample series. (**c**) Arithmetic means of the series' means (in green, as in (b)) and of the sample replication (in orange, as in (a)). (**d**) Scatterplot showing the overall relationship between the mean δ^{18}O and sample replication.

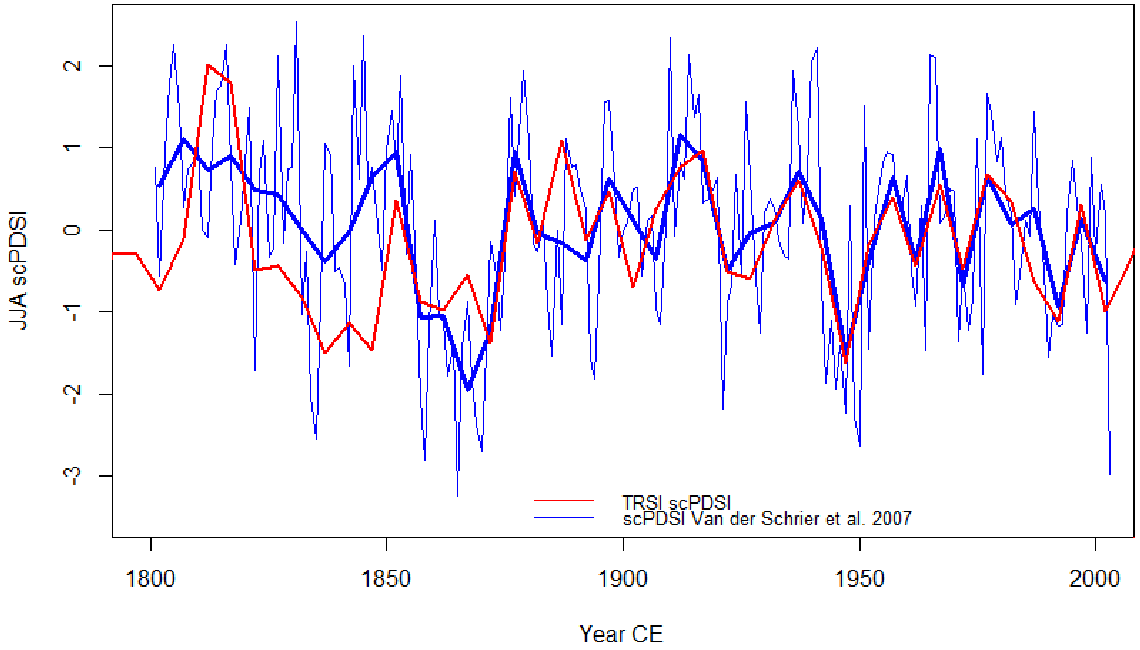

Figure S18. Comparison between an inverse JJA TRSI scPDSI (red line) and a JJA Alpine scPDSI. The JJA TRSI scPDSI with a five-year resolution (in red) and the JJA Alpine scPDSI (*23*) with annual resolution (thin blue) and a five-year mean (thick blue) in the past 200 years.

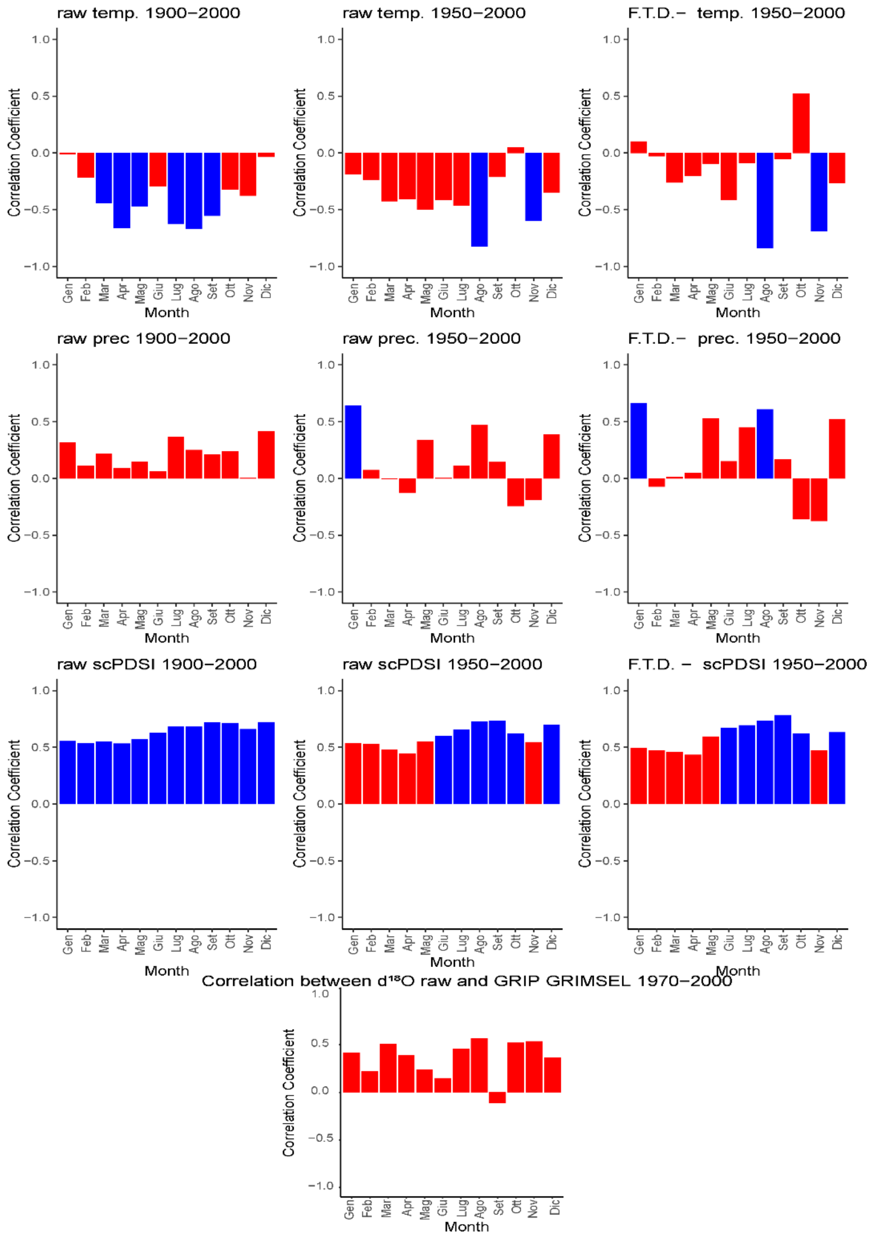

Figure S19. Correlations of the reconstructed scPDSI with monthly climate variables and precipitation isotopes: temperature (first row), precipitation (second row) and scPDSI (third row). The climate parameters are averaged over 46–47° N and 7.55–12.25° E. The correlations are computed using the raw data for 1900–2000 CE (left column) and 1950–2000 (central column), and using the first-time difference for 1950–2000 CE (right column). Non-significant correlations (p >0.05) are shown in red, while significant correlations are shown in blue. The last line is the correlation of the reconstructed tree-ring $\delta^{18}O$ values with monthly values of $\delta^{18}O$ of precipitation, measured at the Grimsel Pass (CH) station for the period 1970–2015, using five-year interpolation. Non-significant correlations (p >0.05) are shown in red, while significant correlations are shown in blue.

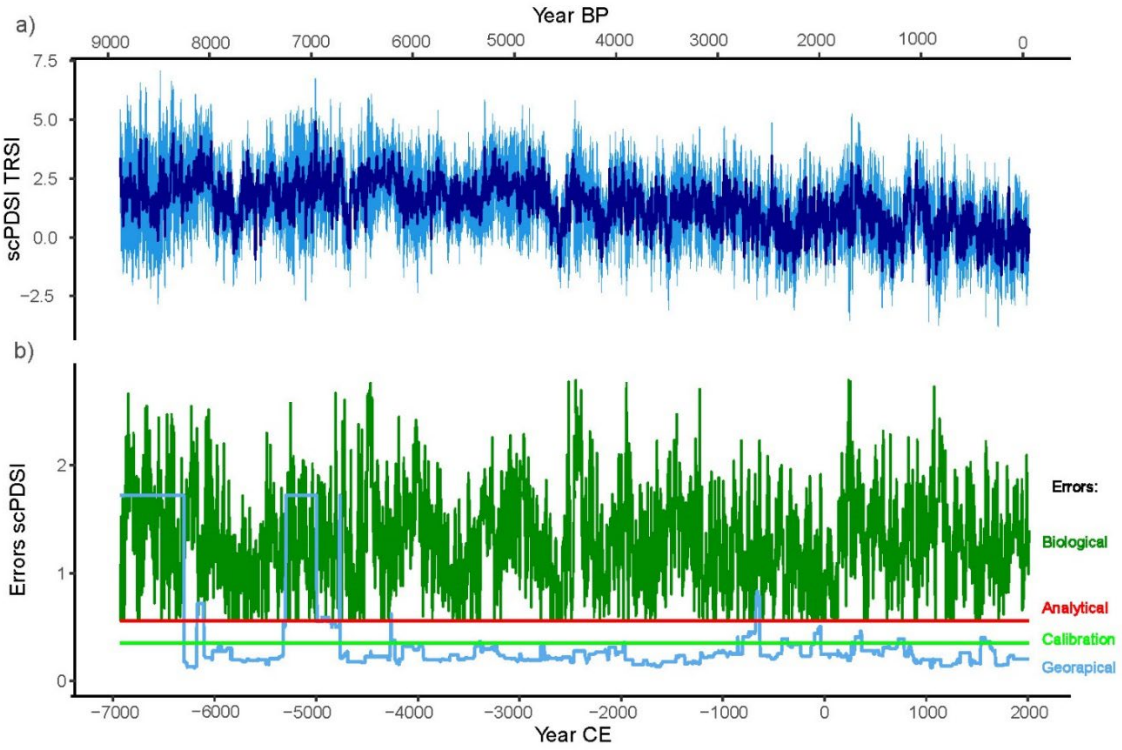

Figure S20. **Different error types of the chronology.** (**a**) scPDSI reconstruction (in blue) and the total error as roots of the sum of the single errors squared (light blue). (**b**) The four errors that were calculated: biological error (in green), analytical error (in red), calibration error (in light green), and geographical error (in light blue).

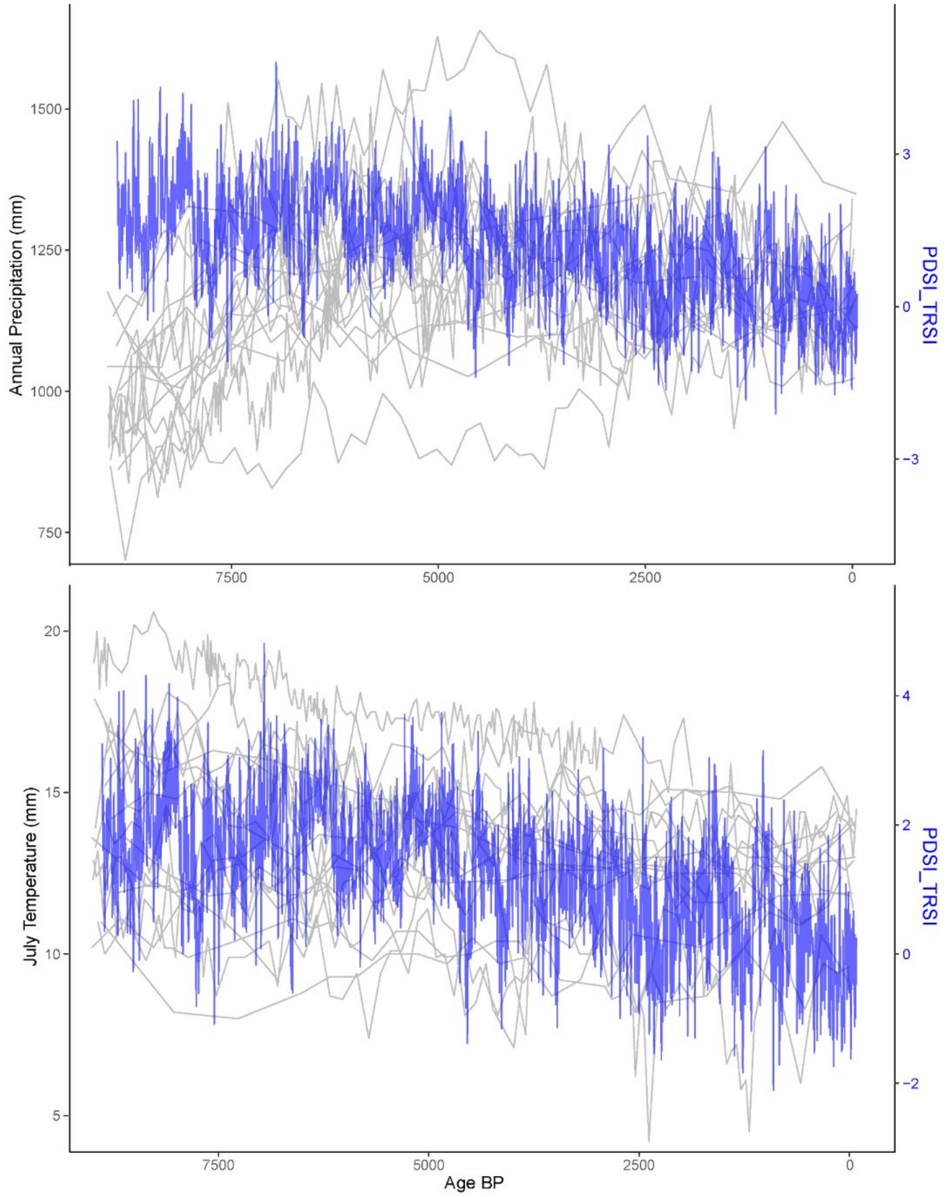

Figure S21. Comparison of the scPDSI TRSI and annual precipitation and summer temperature reconstructions from the Alps. Comparison with 17 reconstructions of July temperatures and annual precipitation from the Alpine region used by Hancock et al (*75*) and the TRSI scPDSI of this study. The reconstructions used are publically available on: https://lipdverse.org/HoloceneHydroclimate/0_7_0/

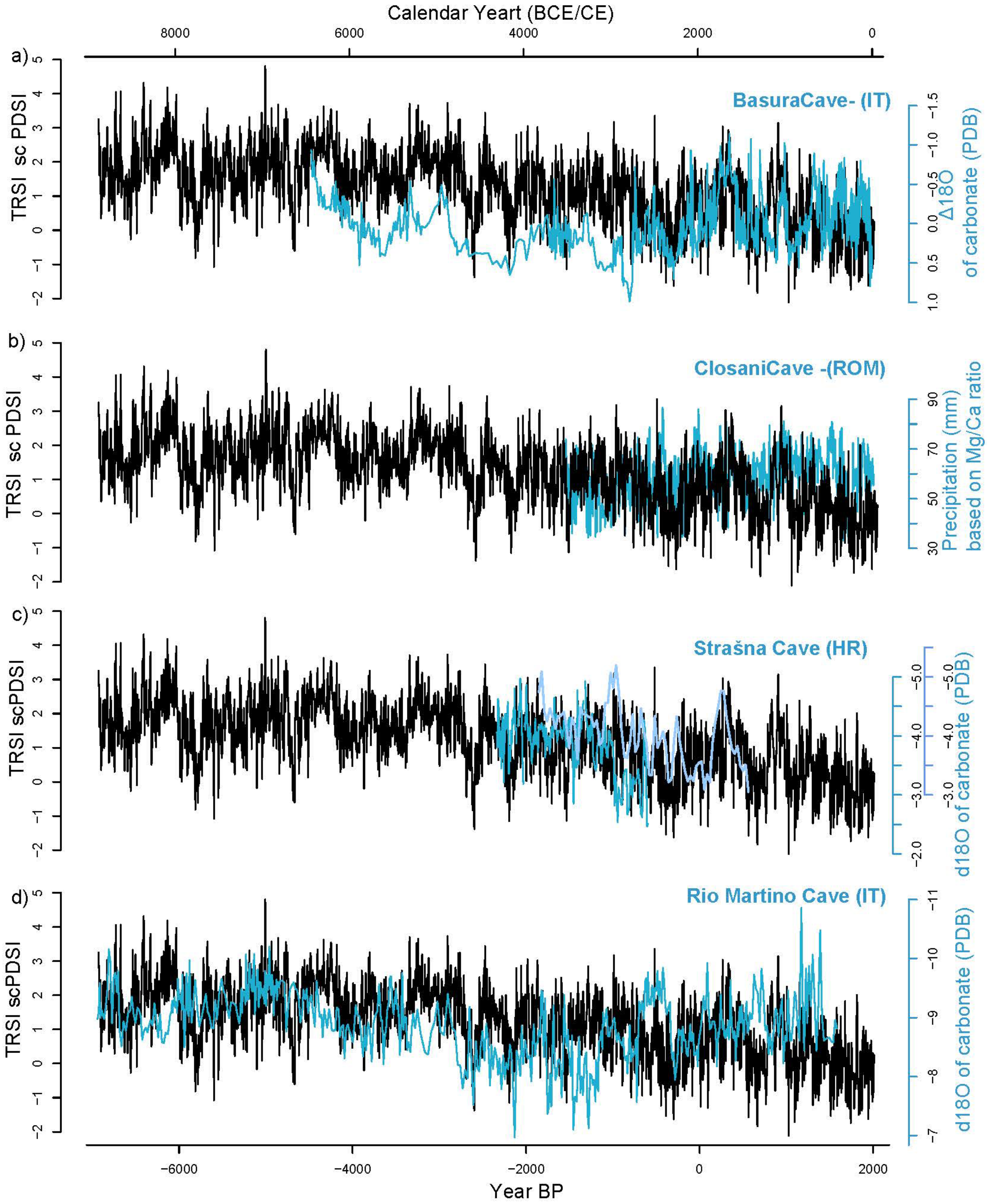

Figure S22. Multi-millennial scPDSI reconstruction from this study (in black) compared with previously published water isotope-based precipitation records from speleothem across Europe (in blue): (**a**) Basura Cave record from Italy (*89*), (**b**) Closani Cave record from Romania (*90*), (**c**) Strašna Cave record from Croatia (*91*), (**d**) Rio Martino Cave record from Italy (*36*). Negative values of the speleothem records indicate drier conditions, and positive values reflect wetter periods. In our reconstruction, negative scPDSI values indicate higher $\delta^{18}O$ values, while positive values indicate lower $\delta^{18}O$ values.

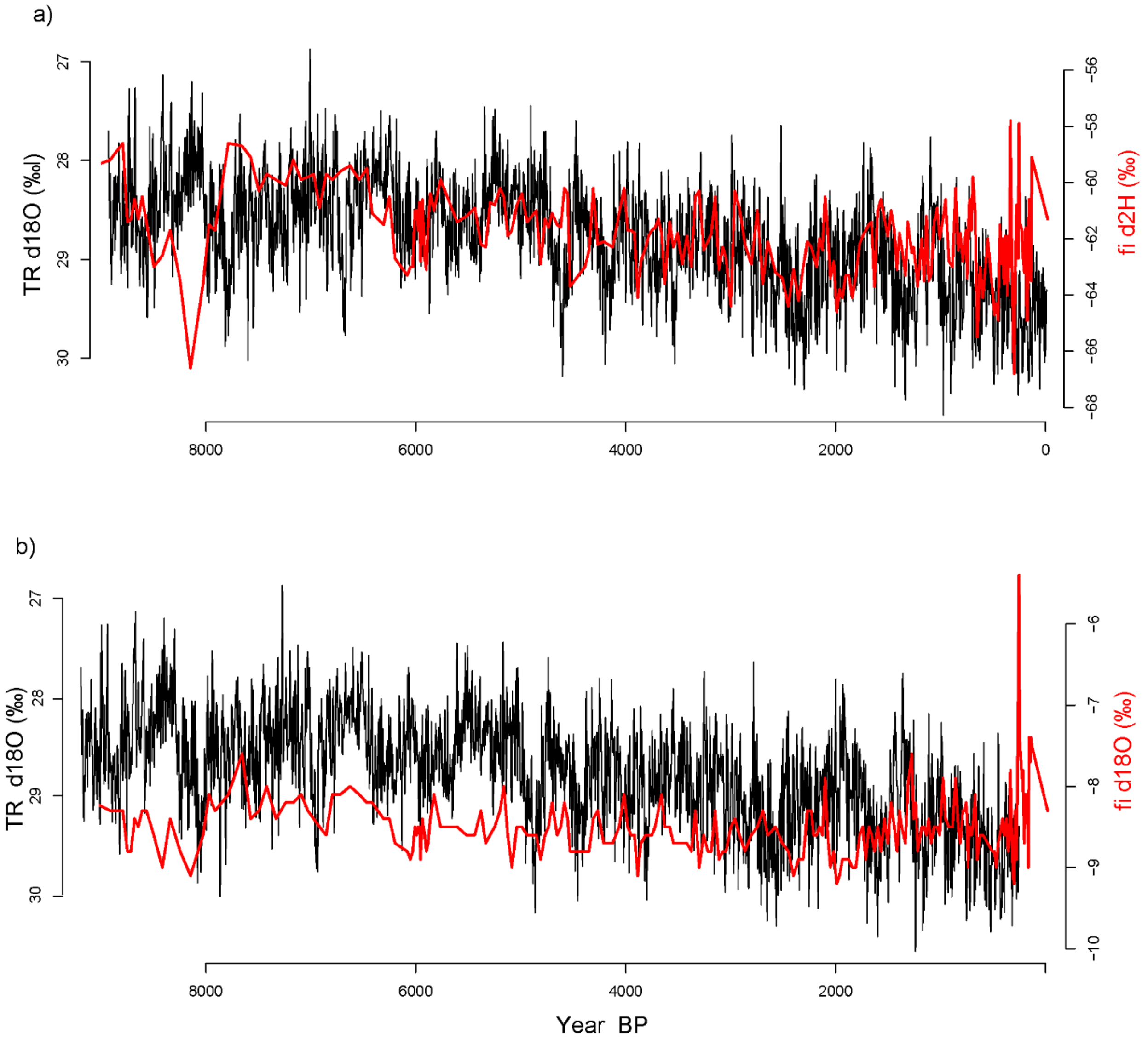

Figure S23. Multi-millennial tree-ring (TR) $\delta^{18}O$ from this study (in black) compared with previously published water isotope from fluid (fi) inclusion from cave Milandre Cave record from Switzerland (in red) (*20*): (**a**) $\delta^{2}H$ from fluid inclusion, (**b**) $\delta^{18}O$ from fluid inclusion. Note the inverse scale of tree-ring $\delta^{18}O$ values.

Supplementary tables S1–4

Table S1. Characteristics of the 29 sampling sites, with number of trees and samples (each sample constitutes of a block of five years).

Site	Place	latitude DMS	longitude DMS	aspect	elevation [m]	sample-type	N. Samples	N. Trees
Ahrntal	Kofler Alm	46.95	12.1	S	2177.5	subfossil	26	1
Ahrntal	Moaralm	47.033333	12.083333	SE	1995	subfossil	567	16
Ahrntal	Starklalm	47.05	12.116667	S	2080	subfossil	87	4
Defereggental	Hirschbichl	46.9	12.25	E	2140	subfossil	36	1
Haslital	Unteraargletscher	46.566667	8.216667	E	1950	subfossil	649	19
Haslital	Unteraargletscher, Rezentproben Nordufer Grimselstausee	46.566667	8.283333	SSE	1977	recent	72	3
Kaunertal	Daunmoränensee	46.883333	10.716667	E	2295	subfossil	581	15
Kaunertal	Gepatschferner	46.866667	10.733333	W	2167.5	subfossil	73	3
Kaunertal	Ombrometer	46.816667	10.7	NE	2147.5	subfossil	190	6
Langtaufers	Sandbichl	46.816667	10.7	NW	2335	subfossil	55	2
Mattertal	Zermatt, Findelengletscher	46.05	7.783333	N	2315	subfossil	80	2
Morteratschgletscher		46.416667	9.933333	W	2045	subfossil	150	3
Ötztal	Ebenalm	47.016667	10.95	NE	2115	subfossil	431	11
Ötztal	Gurgler Alm	46.85	11	W	2175	subfossil	94	3
Ötztal	Gurgler Zirbenwald	46.85	11.016667	NW	2060	subfossil	128	3
Passeier	Timmeltal	46.9	11.133333	S	2117.5	subfossil	117	3
Paznaun	Bielerhöhe	46.916667	10.1	N	2175	subfossil	40	1
Radurschltal	Miseri	46.9	10.616667	N	2252.5	dry-dead	80	2
Rojental		46.8	10.466667	SE	2400	subfossil	103	2
Ultental	Fiechtsee	46.466667	10.833333	N	2110	subfossil	1317	28
Ultental	Weißbrunnalm	46.466667	10.816667	NE	2330	subfossil	80	2
Val d’Hérens	Glacier du Mont Mine	46.033333	7.916667	NNE	1995	subfossil	655	12
Val d’Hérens	Rezentproben Ferpecle	46.066667	7.55	WSW	1965	recent	98	2
Val Roseg	Rezentproben	46.433333	9.85	E	2158.5	recent	146	5
Val Roseg	Tschiervagletscher	46.4	9.883333	NW	2162.5	subfossil	629	14
Vinschgau	Marzoneralm/B	46.583333	10.95	N	2125	subfossil	316	9

Vinschgau	Marzoneral m/C	46.583333	10.95	N	2120	subfossil	126	4
Vinschgau	Marzoneral m/E	46.583333	10.95	N	2125	subfossil	381	13
Vinschgau	Marzoneral m/F	46.583333	10.95	N	2105	subfossil	130	3

Table S2. Crossdating statistics of the tree-ring width series included in the Eastern Alpine Conifer Chronology (EACC, (*15*, *92*)) and utilized for the $\delta^{18}O$ measurements. Calculations were carried out using the program WinTSAP. Overlap: no. of years; Glk.: Gleichläufigkeit; Sign. Glk.: pointer interval Gleichläufigkeit; t-valueBP and t-valueH: t-values after Baillie and Pilcher as well as Hollstein; dating: BCE dates are indicate by "-" and include a year 0.

Sample	Reference chronology	Overlap [n]	Glk. (%]	Sign. Glk. [%]	$t\text{-value}_{BP}$	$t\text{-value}_{H}$	Date first ring	Date last ring
ahmo04	EACC	539	65	86	12.2	14.7	-2133	-1595
ahmo05k	EACC	160	71	84	9.9	9.0	-571	-412
ahmo07	EACC	195	75	90	9.5	11.9	-1789	-1595
ahmo11	EACC	374	64	79	11.0	11.4	-3403	-3030
ahmo19	EACC	210	72	86	10.0	10.4	-641	-432
ahmo25	EACC	213	69	82	9.3	8.5	-3604	-3392
ahmo40	EACC	191	71	81	8.7	8.5	-1270	-1080
ahmo54	EACC	133	67	69	3.7	3.9	-973	-841
ahmo56	EACC	376	61	68	5.4	5.0	-4556	-4181
ahmo59	EACC	270	75	90	16.6	16.2	845	1114
ahmo60	EACC	143	72	82	8.2	10.6	-245	-103
ahmo61	EACC	349	70	79	10.1	10.7	-1151	-803
ahmo63	EACC	345	66	74	11.9	12.0	-1119	-775
ahmo70	EACC	185	78	86	12.3	12.7	117	301
ahmo75	EACC	175	76	90	11.4	12.3	450	624
ahmo81	EACC	259	73	88	11.8	12.2	-1581	-1323
ahst03	EACC	290	76	86	15.1	15.0	307	596
ahst04	EACC	162	60	59	4.5	4.0	713	874
ahst06	EACC	306	63	82	11.3	11.5	-2624	-2319
ahst11	EACC	257	67	84	9.7	9.0	-102	154
bih17	EACC	270	61	76	7.6	8.2	-3975	-3706
eba011	EACC	108	71	85	6.1	6.6	-1235	-1128
eba018	EACC	286	65	81	8.5	9.3	-1512	-1227
eba019	EACC	277	72	89	12.5	13.7	-1675	-1399
eba027	EACC	115	72	82	7.7	9.4	-1363	-1249
eba028	EACC	153	64	82	5.3	6.1	-2502	-2350
eba032	EACC	105	73	90	6.7	7.3	-1237	-1133
eba035	EACC	365	61	70	7.2	7.1	-714	-350
eba101	EACC	316	69	88	11.0	11.4	768	1083
eba111	EACC	620	68	85	17.2	18.0	-4841	-4222
eba118	EACC	113	65	75	3.3	3.6	-1339	-1227
eba124	EACC	443	67	77	13.2	13.2	-6080	-5638

fpcr01	EACC	336	67	79	11.2	11.8	1680	2015
fpcr06	EACC	160	64	80	7.1	7.2	1856	2015
g25	EACC	292	62	84	6.7	6.7	625	916
g48	EACC	427	71	86	11.2	11.7	317	743
g64	EACC	539	67	82	11.0	10.0	157	695
gdm03	EACC	338	75	87	15.5	17.1	-4702	-4365
gdm08	EACC	261	70	89	11.1	13.0	-3938	-3678
gdm101	EACC	403	71	88	15.5	14.9	-5748	-5346
gdm102	EACC	318	72	90	11.9	12.5	-4516	-4199
gdm103k	EACC	368	63	72	8.0	8.3	-6179	-5812
gdm105	EACC	372	65	74	7.6	8.5	-4348	-3977
gdm13	EACC	330	69	84	11.5	13.1	-3274	-2945
gdm18	EACC	289	73	91	14.2	15.1	-3034	-2746
gdm21	EACC	150	72	82	9.9	10.8	-6170	-6021
gdm23	EACC	158	68	82	5.4	6.3	-3020	-2863
gdm26	EACC	126	62	64	3.9	3.9	-2438	-2313
gdm27k	EACC	198	65	75	5.9	6.0	-3817	-3620
gdm32	EACC	254	75	93	13.5	14.4	-2664	-2411
gdm44k	EACC	190	76	93	12.0	11.3	-2881	-2692
gdm46	EACC	252	71	84	10.7	11.1	-3605	-3354
ggua12	EACC	184	70	88	8.3	11.1	-2140	-1957
ggua22	EACC	132	70	85	5.7	7.1	-1090	-959
ggua25	EACC	190	66	78	5.9	5.7	-6286	-6097
gli110	EACC	434	65	78	11.9	11.7	-3562	-3129
gli13	EACC	231	63	79	8.9	8.3	-2843	-2613
gli27	EACC	155	74	93	12.2	11.4	-489	-335
gli35	EACC	192	75	89	12.7	13.1	-2747	-2556
gli40	EACC	303	66	83	8.1	8.5	1089	1391
gli42	EACC	120	63	76	7.0	6.2	-138	-19
gp022	EACC	236	68	83	8.5	7.1	-951	-716
gp025k	EACC	97	66	76	3.8	4.3	1063	1159
gp107	EACC	142	66	91	6.5	6.8	616	757
gp129	EACC	96	65	83	4.3	4.3	-733	-638
gp184k	GP-IA*	62	71	69	3.5	4.7	-798	-737
hib22	EACC	189	64	67	5.8	6.5	-3699	-3511
kofl02k	EACC	135	68	77	5.1	6.4	-3351	-3217
lfs04	EACC	352	69	75	11.8	12.3	-1144	-793
lfs05	EACC	281	73	89	12.3	12.5	-506	-226
mazb01	EACC	184	63	73	7.4	6.4	1438	1621
mazb12	EACC	329	70	85	14.0	13.7	-5984	-5656
mazb24	EACC	303	71	84	14.1	14.8	-2273	-1971
mazb52k	EACC	425	69	88	17.0	18.5	-976	-552
mazb53	EACC	311	72	84	14.0	15.0	-827	-517
mazb62	EACC	302	64	83	8.0	7.9	-781	-480
mazb63	EACC	365	74	85	16.8	17.3	-1667	-1303
mazb65	EACC	148	76	81	11.6	10.0	-1257	-1110
mazb72	EACC	313	74	86	14.0	13.4	-816	-504
mazc01k	EACC	370	78	89	18.2	15.2	-321	48
mazc07k	EACC	432	79	95	25.4	25.4	-245	186

mazc14	EACC	253	79	88	16.0	16.1	1536	1788
mazc15	EACC	177	73	90	14.4	14.5	1517	1693
maze04k	EACC	116	62	68	4.8	4.7	1548	1663
maze06	EACC	311	76	91	15.9	15.2	934	1244
maze09	EACC	166	60	69	4.1	3.8	-1464	-1299
maze20	EACC	219	75	93	13.1	13.5	671	889
maze22	EACC	414	60	60	6.9	6.7	-1049	-636
maze24	EACC	283	72	83	12.8	13.2	123	405
maze32	EACC	252	63	87	9.6	10.7	1336	1587
maze34k	EACC	112	70	88	7.5	8.1	-1153	-1042
maze52	EACC	274	67	80	8.0	7.6	-108	165
maze62k	EACC	174	66	74	4.9	4.1	1604	1777
maze73	EACC	117	68	84	5.0	5.1	-1094	-978
maze75	EACC	409	71	89	19.9	16.4	200	608
maze77	EACC	574	76	87	22.8	21.2	-203	370
mazf04	EACC	276	67	88	11.9	12.0	-717	-442
mazf08	EACC	326	71	87	12.3	12.6	-686	-361
mazf10	EACC	381	69	83	13.4	13.3	-1006	-626
mis095	EACC	393	73	92	14.6	16.0	1495	1887
mis164	EACC	207	70	87	8.1	8.1	1275	1481
mm11-5	EACC	553	69	87	18.5	22.0	-6861	-6309
mm602	EACC	258	68	79	9.7	9.8	-6450	-6193
mm605	EACC	743	70	86	21.0	20.6	-6955	-6213
mm606	EACC	649	62	74	10.1	9.8	-6881	-6233
mm608k	EACC	190	66	80	8.0	9.3	-6386	-6197
mm619	EACC	558	72	92	18.7	19.0	-6935	-6378
mm624k	EACC	164	64	62	4.0	3.3	-6331	-6168
mm626	EACC	143	63	71	3.4	2.8	-6343	-6201
mm633	EACC	369	63	77	12.8	13.3	-6781	-6413
mm903	EACC	311	69	83	7.5	8.8	-6559	-6249
mm907k	EACC	239	72	85	10.1	10.3	-5497	-5259
mm914	EACC	197	79	95	16.9	16.3	1076	1272
mort05k	EACC	225	74	92	11.9	11.8	1272	1496
mort06	EACC	226	66	86	9.9	9.5	852	1077
mort07	EACC	433	71	85	16.0	16.3	1008	1440
rt04	EACC	265	65	79	10.5	11.6	-3458	-3194
rt06	EACC	327	64	68	9.3	9.4	-6300	-5974
tah38	EACC	297	68	81	11.7	10.2	-371	-75
tah39	EACC	231	67	84	7.4	8.2	498	728
tah41	EACC	236	64	75	7.0	5.9	147	382
tsc134k	EACC	270	65	76	7.9	7.6	-4188	-3919
tsc147	EACC	207	65	77	7.9	7.1	-4945	-4739
tsc154	EACC	206	69	84	8.2	8.5	-4948	-4743
tsc157k	EACC	492	58	67	5.4	5.6	-5494	-5003
tsc160	EACC	596	66	79	9.4	9.6	-5474	-4879
tsc173k	EACC	302	67	79	7.7	8.2	-5001	-4700
tsc178	EACC	155	66	82	6.9	7.1	-4875	-4721
tsc182	EACC	431	64	69	9.0	8.9	-5336	-4906
tsc189	EACC	442	68	73	12.0	9.4	-5367	-4926

tsc211	EACC	144	66	85	7.7	7.8	-4207	-4064
tsc221	EACC	117	63	74	4.5	3.7	-4012	-3896
tsc223	EACC	190	60	70	5.4	4.6	-4156	-3967
ua119	EACC	246	69	74	7.1	7.5	-2322	-2077
ua126	EACC	114	56	3.1	3.4	19	-3789	-3676
ua128	EACC	133	68	78	6.8	6.8	-1910	-1778
ua134	EACC	237	72	87	12.9	10.9	-2715	-2479
ua174k	EACC	294	71	79	12.9	13.8	-1752	-1459
ua178	EAAC[§]	89	67	70	3.1	3.5	-2340	-2252
ua181	EACC	103	64	88	5.8	7.1	-2583	-2481
ua210	EAAC[#]	134	66	70	5.4	4.5	-1351	-1218
ua211	EACC	336	67	78	9.1	8.7	-3045	-2710
ua344	EACC	89	70	87	6.9	6.7	-6109	-6021
ua345	EACC	191	72	81	11.0	11.9	-5751	-5561
ua346k	EACC	369	72	86	17.2	17.8	-5705	-5337
ua401	EACC	244	76	90	11.9	12.6	-2707	-2464
ua405	EACC	417	71	86	14.1	13.7	-4225	-3809
ua408	EACC	92	70	88	7.2	7.1	-1384	-1293
ua413	EACC	198	65	77	7.2	8.3	-2659	-2462
ua414	EACC	93	68	82	5.4	6.1	-3869	-3777
ua423	EACC	218	74	88	12.5	11.7	-5865	-5648
ua437k	EACC	155	58	63	5.4	4.6	-4676	-4522
uazr01	EACC	175	68	86	7.9	8.9	1841	2015
uazr02	EACC	230	69	82	9.6	10.2	1786	2015
ulfi004	EACC	181	68	72	8.4	8.1	-2225	-2045
ulfi005	EACC	251	66	79	7.7	8.2	-3222	-2972
ulfi010	EACC	222	69	77	8.4	8.4	-2948	-2727
ulfi012	EACC	274	66	84	8.0	8.3	-3181	-2908
ulfi022	EACC	258	72	87	13.1	11.0	554	811
ulfi034	EACC	372	71	81	13.1	9.8	-3732	-3361
ulfi037	EACC	292	62	79	6.2	6.1	-4556	-4265
ulfi038	EACC	97	77	92	8.6	8.7	-1206	-1110
ulfi039k	EACC	238	65	77	6.0	6.8	-4765	-4528
ulfi044	EACC	336	73	86	14.9	15.6	-5698	-5363
ulfi047	EACC	524	71	87	21.8	21.6	-4120	-3597
ulfi053	EACC	585	64	84	13.0	13.8	-5053	-4469
ulfi055	EACC	477	64	85	12.3	9.9	593	1069
ulfi056	EACC	324	57	66	7.3	6.9	-1976	-1653
ulfi057	EACC	189	66	77	5.0	4.6	-4744	-4556
ulfi063	EACC	360	71	86	14.6	14.3	-2477	-2118
ulfi066	EACC	457	71	82	15.7	15.8	-1863	-1407
ulfi067	EACC	241	67	79	12.7	11.9	-530	-290
ulfi069	EACC	329	66	76	8.1	8.4	-2477	-2149
ulfi071	EACC	241	74	83	13.2	14.1	-697	-457
ulfi079	EACC	227	67	78	10.8	10.9	-457	-231
ulfi084	EACC	166	70	88	9.9	9.7	1024	1189
ulfi088	EACC	366	70	86	15.4	15.9	516	881
ulfi090	EACC	216	60	70	3.8	2.8	1249	1464
ulfi099	EACC	251	70	88	13.8	13.8	1437	1687

ulfi104	EACC	368	70	79	13.2	13.3	-289	78
ulfi107	EACC	631	70	86	19.6	18.9	-4007	-3377
ulfi109	EACC	558	70	83	16.5	15.0	-6027	-5470
uwba02	EACC	405	72	79	13.5	15.5	-6108	-5704
uwba03	EACC	487	66	80	13.8	12.0	-5520	-5034
vrr03	EACC	196	62	77	6.8	5.5	1819	2014
vrr05	EACC	255	64	84	8.7	9.0	1760	2014
vrr07	EACC	150	70	78	5.9	6.5	1865	2014
zer1001	EACC	436	65	86	14.3	13.5	1165	1600
zer1401	EACC	410	68	86	15.2	14.6	936	1345

* site chronology Gepatschferner – Iron Age, § EACC *Larix decidua* only chronology, # EACC *Pinus cembra* only chronology.

Table S3. Statistical information of the calibration model (1901–2018), the verification period (1850–1900) and the full period (1850–2000). Each column represents a different measure of the relationship between the climate target and proxy variable along with, where appropriate, a correlation Pearson correlation factor (r), a coefficient of determination (R2), a p-value (*p*), reduction of error (RE), coefficient of efficiency (CE), Durbin-Watson statistic (DW), and Durbin's h-statistic (DE).

Time window	r	R2	*p*	RE	CE	DW	DE	n
1901-2000	0.85	0.72	2.015e-06	0.72	0.72	2.42	0.9	20
1850-1900	0.76	0.57	0.010	0.57	0.57	2.65	0.98	10
1850-2000	0.79	0.63	1.645e-07	0.63	0.63	2.03	0.98	30

Table S4. Comparison of our JJA scPDSI reconstruction with warm-season precipitation-related and temperature records. Paleoclimatic records from Europe. Pearson correlation factor (r) values were computed from 1500 CE to the present. Abbreviations: TR, tree-ring; MXD, maximum latewood density; scPDSI, self-calibrated Palmer Drought Severity Index; SPEI, Standardized Precipitation-Evapotranspiration Index, original, unfiltered; Precip., precipitation; Temp., temperature. GHD grape harvest starting dates. SPI, Standardized Precipitation Index.

	Büntgen (*2*)	Cook (*71*)	Nagavciuc (*93*)	Labuhn (*94*)	Büntgen (*95*)	Luterbacher (*46*)	Wetter Pfister (*72*)	Casty (*73*)	KlippeRl (*96*)	Casty (*73*)
Proxy	TR $\delta^{18}O$ & $\delta^{13}C$	TRW	TR $\delta^{18}O$	TR $\delta^{18}O$	TRW	TRW and MXD	GHD	Instrumental and document	TRW	Instrumental and document
Parameter	scPDSI	scPDSI	SPEI	SPEI	Temp.	Temp.	Temp.	Temp.	SPI	Precip.
Region	Central Europe	Alps	Eastern Europe	France	Central Europe	Europe	Switzerland	Alps	Balkan Peninsula	Alps
Season	JJA	JJA	JJA	JJA	JJA	JJA	AMJJ	JJA	JJ	JJA
r 5ry interpolation	0.18	0.19	0.10	0.11	-0.20	-0.11	-0.1	-0.11	-0.16	0.20
r 50yr Mean	0.49	0.53	0.07	0.02	-0.10	-0.31	-0.42	-0.30	-0.30	0.23

REFERENCES AND NOTES

1. B. I. Cook, J. S. Mankin, K. Marvel, A. P. Williams, J. E. Smerdon, K. J. Anchukaitis, Twenty-first century drought projections in the CMIP6 forcing scenarios. *Earth's Future* **8**, e2019EF001461 (2020).

2. U. Büntgen, O. Urban, P. J. Krusic, M. Rybníček, T. Kolář, T. Kyncl, A. Ač, E. Koňasová, J. Čáslavskỳ, J. Esper, S. Wagner, M. Saurer, W. Tegel, P. Dobrovolný, P. Cherubini, F. Reinig, M. Trnka, Recent European drought extremes beyond Common era background variability. *Nat. Geosci.* **14**, 190–196 (2021).

3. V. Hari, O. Rakovec, Y. Markonis, M. Hanel, R. Kumar, Increased future occurrences of the exceptional 2018–2019 Central European drought under global warming. *Sci. Rep.* **10**, 12207 (2020).

4. S. C. Scherrer, M. Hirschi, C. Spirig, F. Maurer, S. Kotlarski, Trends and drivers of recent summer drying in Switzerland. *Environ. Res. Commun.* **4**, 025004 (2022).

5. J. Esper, M. Torbenson, U. Büntgen, 2023 summer warmth unparalleled over the past 2,000 years. *Nature* **631**, 94–97 (2024).

6. F. C. Ljungqvist, P. J. Krusic, H. S. Sundqvist, E. Zorita, G. Brattström, D. Frank, Northern Hemisphere hydroclimate variability over the past twelve centuries. *Nature* **532**, 94–98 (2016).

7. C. M. Brierley, A. Zhao, S. P. Harrison, P. Braconnot, C. J. R. Williams, D. J. R. Thornalley, X. Shi, J.-Y. Peterschmitt, R. Ohgaito, D. S. Kaufman, M. Kageyama, J. C. Hargreaves, M. P. Erb, J. Emile-Geay, R. D'Agostino, D. Chandan, M. Carré, P. J. Bartlein, W. Zheng, Z. Zhang, Q. Zhang, H. Yang, E. M. Volodin, R. A. Tomas, C. Routson, W. R. Peltier, B. Otto-Bliesner, P. A. Morozova, N. P. McKay, G. Lohmann, A. N. Legrande, C. Guo, J. Cao, E. Brady, J. D. Annan, A. Abe-Ouchi, Large-scale features and evaluation of the PMIP4-CMIP6 *midHolocene* simulations. *Clim. Past* **16**, 1847–1872 (2020).

8. H. Essell, P. J. Krusic, J. Esper, S. Wagner, P. Braconnot, J. Jungclaus, F. Muschitiello, C. Oppenheimer, U. Büntgen, A frequency-optimised temperature record for the Holocene. *Environ. Res. Lett.* **18**, 114022 (2023).

9. U. Büntgen, Scrutinizing tree-ring parameters for Holocene climate reconstructions. *WIREs Clim. Change* **13**, e778 (2022).

10. D. S. Kaufman, E. Broadman, Revisiting the Holocene global temperature conundrum. *Nature* **614**, 425–435 (2023).

11. E. R. Cook, K. R. Briffa, D. M. Meko, D. A. Graybill, G. Funkhouser, The'segment length curse'in long tree-ring chronology development for palaeoclimatic studies. *Holocene* **5**, 229–237 (1995).

12. E. Martínez-Sancho, L. A. Cernusak, P. Fonti, A. Gregori, B. Ullrich, E. G. Pannatier, A. Gessler, M. M. Lehmann, M. Saurer, K. Treydte, Unenriched xylem water contribution during cellulose synthesis influenced by atmospheric demand governs the intra-annual tree-ring $\delta^{18}O$ signature. *New Phytol.* **240**, 1743–1757 (2023).

13. T. Nakatsuka, M. Sano, Z. Li, C. Xu, A. Tsushima, Y. Shigeoka, K. Sho, K. Ohnishi, M. Sakamoto, H. Ozaki, N. Higami, N. Nakao, M. Yokoyama, T. Mitsutani, A 2600-year summer climate reconstruction in central Japan by integrating tree-ring stable oxygen and hydrogen isotopes. *Clim. Past* **16**, 2153–2172 (2020).

14. B. Yang, C. Qin, A. Bräuning, T. J. Osborn, V. Trouet, F. C. Ljungqvist, J. Esper, L. Schneider, J. Grießinger, U. Büntgen, S. Rossi, G. Dong, M. Yan, L. Ning, J. Wang, X. Wang, S. Wang, J. Luterbacher, E. R. Cook, N. C. Stenseth, Long-term decrease in Asian monsoon rainfall and abrupt climate change events over the past 6,700 years. *Proc. Natl. Acad. Sci. U.S.A.* **118**, e2102007118 (2021).

15. K. Nicolussi, M. Kaufmann, T. M. Melvin, J. Van Der Plicht, P. Schießling, A. Thurner, A 9111 year long conifer tree-ring chronology for the European Alps: A base for environmental and climatic investigations. *Holocene* **19**, 909–920 (2009).

16. T. Arosio, M. M. Ziehmer, K. Nicolussi, C. Schlüchter, M. Leuenberger, Alpine Holocene tree-ring dataset: Age-related trends in the stable isotopes of cellulose show species-specific patterns. *Biogeosciences* **17**, 4871–4882 (2020).

17. P. Hafner, I. Robertson, D. McCarroll, N. J. Loader, M. Gagen, R. J. Bale, H. Jungner, E. Sonninen, E. Hilasvuori, T. Levanič, Climate signals in the ring widths and stable carbon, hydrogen and oxygen isotopic composition of *Larix decidua* growing at the forest limit in the southeastern European Alps. *Trees* **25**, 1141–1154 (2011).

18. K. Treydte, S. Boda, E. G. Pannatier, P. Fonti, D. Frank, B. Ullrich, M. Saurer, R. Siegwolf, G. Battipaglia, W. Werner, A. Gessler, Seasonal transfer of oxygen isotopes from precipitation and soil to the tree ring: Source water versus needle water enrichment. *New Phytol.* **202**, 772–783 (2014).

19. H. Sodemann, E. Zubler, Seasonal and inter-annual variability of the moisture sources for Alpine precipitation during 1995–2002. *Int. J. Climatol.* **30**, 947–961 (2010).

20. S. Affolter, A. Häuselmann, D. Fleitmann, R. L. Edwards, H. Cheng, M. Leuenberger, Central Europe temperature constrained by speleothem fluid inclusion water isotopes over the past 14,000 years. *Sci. Adv.* **5**, eaav3809 (2019).

21. B. S. Lecavalier, D. A. Fisher, G. A. Milne, B. M. Vinther, L. Tarasov, P. Huybrechts, D. Lacelle, B. Main, J. Zheng, J. Bourgeois, A. S. Dyke, High Arctic Holocene temperature record from the Agassiz ice cap and Greenland ice sheet evolution. *Proc. Natl. Acad. Sci. U.S.A.* **114**, 5952–5957 (2017).

22. N. Wells, S. Goddard, M. J. Hayes, A self-calibrating Palmer drought severity index. *J. Climate* **17**, 2335–2351 (2004).

23. G. Van der Schrier, D. Efthymiadis, K. R. Briffa, P. D. Jones, European Alpine moisture variability for 1800–2003. *Int. J. Climatol.* **27**, 415–427 (2007).

24. M. B. Freund, G. Helle, D. F. Balting, N. Ballis, G. H. Schleser, U. Cubasch, European tree-ring isotopes indicate unusual recent hydroclimate. *Commun. Earth Environ.* **4**, 26 (2023).

25. V. Nagavciuc, M. Ionita, A. Perşoiu, I. Popa, N. J. Loader, D. McCarroll, Stable oxygen isotopes in Romanian oak tree rings record summer droughts and associated large-scale circulation patterns over Europe. *Climate Dynam.* **52**, 6557–6568 (2019).

26. B. A. S. Davis, S. Brewer, Orbital forcing and role of the latitudinal insolation/temperature gradient. *Climate Dynam.* **32**, 143–165 (2009).

27. K. Nicolussi, M. Kaufmann, G. Patzelt, J. Plicht van der, A. Thurner, Holocene tree-line variability in the Kauner Valley, Central Eastern Alps, indicated by dendrochronological analysis of living trees and subfossil logs. *Veget. Hist. Archaeobot.* **14**, 221–234 (2005).

28. T. M. Shanahan, N. P. McKay, K. A. Hughen, J. T. Overpeck, B. Otto-Bliesner, C. W. Heil, J. King, C. A. Scholz, J. Peck, The time-transgressive termination of the African Humid Period. *Nat. Geosci.* **8**, 140–144 (2015).

29. M. Finné, K. Holmgren, H. S. Sundqvist, E. Weiberg, M. Lindblom, Climate in the eastern Mediterranean, and adjacent regions, during the past 6000 years–A review. *J. Archaeol. Sci.* **38**, 3153–3173 (2011).

30. M. Le Roy, S. Ivy-Ochs, K. Nicolussi, G. Monegato, J. M. Reitner, R. R. Colucci, A. Ribolini, M. Spagnolo, M. Stoffel, "Chapter 20: Holocene glacier variations in the Alps" in *European Glacial Landscapes: The Holocene* (Elsevier, 2024), pp. 367–418; https://doi.org/10.1016/B978-0-323-99712-6.00018-0.

31. S. C. Sherwood, R. Roca, T. M. Weckwerth, N. G. Andronova, Tropospheric water vapor, convection, and climate. *Rev. Geophys.* **48**, RG2001 (2010).

32. B. Bisselink, A. J. Dolman, Precipitation recycling: Moisture sources over Europe using ERA-40 data. *J. Hydrometeorol.* **9**, 1073–1083 (2008).

33. G. Bond, W. Showers, M. Cheseby, R. Lotti, P. Almasi, P. DeMenocal, P. Priore, H. Cullen, I. Hajdas, G. Bonani, A pervasive millennial-scale cycle in North Atlantic Holocene and glacial climates. *Science* **278**, 1257–1266 (1997).

34. K. Nicolussi, C. Schlüchter, The 8.2 ka event—Calendar-dated glacier response in the Alps. *Geology* **40**, 819–822 (2012).

35. M. Magny, Holocene climate variability as reflected by mid-European lake-level fluctuations and its probable impact on prehistoric human settlements. *Quat. Int.* **113**, 65–79 (2004).

36. E. Regattieri, G. Zanchetta, I. Isola, E. Zanella, R. N. Drysdale, J. C. Hellstrom, A. Zerboni, L. Dallai, E. Tema, L. Lanci, E. Costa, F. Magrì, Holocene Critical Zone dynamics in an Alpine catchment inferred from a speleothem multiproxy record: Disentangling climate and human influences. *Sci. Rep.* **9**, 17829 (2019).

37. E. R. Thomas, E. W. Wolff, R. Mulvaney, J. P. Steffensen, S. J. Johnsen, C. Arrowsmith, J. W. C. White, B. Vaughn, T. Popp, The 8.2 ka event from Greenland ice cores. *Quat. Sci. Rev.* **26**, 70–81 (2007).

38. J. Fohlmeister, A. Schröder-Ritzrau, D. Scholz, C. Spötl, D. F. C. Riechelmann, M. Mudelsee, A. Wackerbarth, A. Gerdes, S. Riechelmann, A. Immenhauser, D. K. Richter, A. Mangini, Bunker Cave stalagmites: An archive for central European Holocene climate variability. *Clim. Past* **8**, 1751–1764 (2012).

39. M. Liu, Y. Shen, P. González-Sampériz, G. Gil-Romera, C. J. F. ter Braak, I. C. Prentice, S. P. Harrison, Holocene climates of the Iberian Peninsula: Pollen-based reconstructions of changes in the west–east gradient of temperature and moisture. *Clim. Past* **19**, 803–834 (2023).

40. J. C. H. Chiang, C. M. Bitz, Influence of high latitude ice cover on the marine Intertropical Convergence Zone. *Climate Dynam.* **25**, 477–496 (2005).

41. H. Hercman, M. Gąsiorowski, J. Pawlak, M. Błaszczyk, M. Gradziński, Š. Matoušková, P. Zawidzki, P. Bella, Atmospheric circulation and the differentiation of precipitation sources during the Holocene inferred from five stalagmite records from Demänová Cave System (Central Europe). *Holocene* **30**, 834–846 (2020).

42. M. Magny, J.-L. de Beaulieu, R. Drescher-Schneider, B. Vannière, A.-V. Walter-Simonnet, Y. Miras, L. Millet, G. Bossuet, O. Peyron, E. Brugiapaglia, A. Leroux, Holocene climate changes in the central Mediterranean as recorded by lake-level fluctuations at Lake Accesa (Tuscany, Italy). *Quat. Sci. Rev.* **26**, 1736–1758 (2007).

43. F. C. Ljungqvist, A new reconstruction of temperature variability in the extra-tropical Northern Hemisphere during the last two millennia. *Geogr. Ann. Ser. B* **92**, 339–351 (2010).

44. U. Büntgen, V. S. Myglan, F. C. Ljungqvist, M. McCormick, N. Di Cosmo, M. Sigl, J. Jungclaus, S. Wagner, P. J. Krusic, J. Esper, J. O. Kaplan, M. A. C. de Vaan, J. Luterbacher, L. Wacker, W. Tegel, A. V. Kirdyanov, Cooling and societal change during the Late Antique Little Ice Age from 536 to around 660 AD. *Nat. Geosci.* **9**, 231–236 (2016).

45. W. Tegel, A. Seim, G. Skiadaresis, F. C. Ljungqvist, H.-P. Kahle, A. Land, B. Muigg, K. Nicolussi, U. Büntgen, Higher groundwater levels in western Europe characterize warm periods in the Common era. *Sci. Rep.* **10**, 16284 (2020).

46. J. Luterbacher, J. P. Werner, J. E. Smerdon, L. Fernández-Donado, F. J. González-Rouco, D. Barriopedro, F. C. Ljungqvist, U. Büntgen, E. Zorita, S. Wagner, J. Esper, D. McCarroll, A. Toreti, D. Frank, J. H. Jungclaus, M. Barriendos, C. Bertolin, O. Bothe, R. Brázdil, D. Camuffo, P. Dobrovolný, M. Gagen, E. García-Bustamante, Q. Ge, J. J. Gómez-Navarro, J. Guiot, Z. Hao, G. C. Hegerl, K. Holmgren, V. V. Klimenko, J. Martín-Chivelet, C. Pfister, N. Roberts, A. Schindler, A. Schurer, O. Solomina, L. von Gunten, E. Wahl, H. Wanner, O. Wetter, E. Xoplaki,

N. Yuan, D. Zanchettin, H. Zhang, C. Zerefos, European summer temperatures since Roman times. *Environ. Res. Lett.* **11**, 024001 (2016).

47. M. Frachetti, N. Di Cosmo, J. Esper, L. Khalidi, F. Mauelshagen, C. Oppenheimer, E. Rohland, U. Büntgen, The dahliagram: An interdisciplinary tool for investigation, visualization, and communication of past human-environmental interaction. *Sci. Adv.* **9**, eadj3142 (2023).

48. H. Wanner, J. Beer, J. Bütikofer, T. J. Crowley, U. Cubasch, J. Flückiger, H. Goosse, M. Grosjean, F. Joos, J. O. Kaplan, M. Küttel, S. A. Müller, I. C. Prentice, O. Solomina, T. F. Stocker, P. Tarasov, M. Wagner, M. Widmann, Mid-to Late Holocene climate change: An overview. *Quat. Sci. Rev.* **27**, 1791–1828 (2008).

49. I. Auer, R. Böhm, A. Jurkovic, W. Lipa, A. Orlik, R. Potzmann, W. Schöner, M. Ungersböck, C. Matulla, K. Briffa, P. Jones, D. Efthymiadis, M. Brunetti, T. Nanni, M. Maugeri, L. Mercalli, O. Mestre, J. M. Moisselin, M. Begert, G. Müller-Westermeier, V. Kveton, O. Bochnicek, P. Stastny, M. Lapin, S. Szalai, T. Szentimrey, T. Cegnar, M. Dolinar, M. Gajic-Capka, K. Zaninovic, Z. Majstorovic, E. Nieplova, HISTALP—Historical instrumental climatological surface time series of the Greater Alpine Region. *Int. J. Climatol.* **27**, 17–46 (2007).

50. H. Wanner, R. Rickli, E. Salvisberg, C. Schmutz, M. Schüepp, Global climate change and variability and its influence on Alpine climate—Concepts and observations. *Theor. Appl. Climatol.* **58**, 221–243 (1997).

51. U. Büntgen, L. Wacker, K. Nicolussi, M. Sigl, D. Güttler, W. Tegel, P. J. Krusic, J. Esper, Extraterrestrial confirmation of tree-ring dating. *Nat. Clim. Change* **4**, 404–405 (2014).

52. U. Büntgen, L. Wacker, J. Galvan, S. Arnold, D. Arseneault, M. Baillie, J. Beer, M. Bernabei, N. Bleicher, G. Boswijk, A. Bräuning, M. Carrer, F. C. Ljungqvist, P. Cherubini, M. Christl, D. A. Christie, P. W. Clark, E. R. Cook, R. D'Arrigo, N. Davi, Ó. Eggertsson, J. Esper, A. M. Fowler, Z. Gedalof, F. Gennaretti, J. Grießinger, H. Grissino-Mayer, H. Grudd, B. E. Gunnarson, R. Hantemirov, F. Herzig, A. Hessl, K.-U. Heussner, A. J. T. Jull, V. Kukarskih, A. Kirdyanov, T. Kolář, P. J. Krusic, T. Kyncl, A. Lara, C. L. Quesne, H. W. Linderholm, N. J. Loader, B.

Luckman, F. Miyake, V. S. Myglan, K. Nicolussi, C. Oppenheimer, J. Palmer, I. Panyushkina, N. Pederson, M. Rybníček, F. H. Schweingruber, A. Seim, M. Sigl, O. Churakova, J. H. Speer, H.-A. Synal, W. Tegel, K. Treydte, R. Villalba, G. Wiles, R. Wilson, L. J. Winship, J. Wunder, B. Yang, G. H. F. Young, Tree rings reveal globally coherent signature of cosmogenic radiocarbon events in 774 and 993 CE. *Nat. Commun.* **9**, 3605 (2018).

53. F. Miyake, I. P. Panyushkina, A. J. T. Jull, F. Adolphi, N. Brehm, S. Helama, K. Kanzawa, T. Moriya, R. Muscheler, K. Nicolussi, M. Oinonen, M. Salzer, M. Takeyama, F. Tokanai, L. Wacker, A single-year cosmic ray event at 5410 BCE registered in 14C of tree rings. *Geophys. Res. Lett.* **48**, e2021GL093419 (2021).

54. N. Brehm, M. Christl, T. D. J. Knowles, E. Casanova, R. P. Evershed, F. Adolphi, R. Muscheler, H.-A. Synal, F. Mekhaldi, C. I. Paleari, H.-H. Leuschner, A. Bayliss, K. Nicolussi, T. Pichler, C. Schlüchter, C. L. Pearson, M. W. Salzer, P. Fonti, D. Nievergelt, R. Hantemirov, D. M. Brown, I. Usoskin, L. Wacker, Tree-rings reveal two strong solar proton events in 7176 and 5259 BCE. *Nat. Commun.* **13**, 1196 (2022).

55. T. Arosio, M. M. Ziehmer-Wenz, K. Nicolussi, C. Schlüchter, M. Leuenberger, Larch cellulose shows significantly depleted hydrogen isotope values with respect to evergreen conifers in contrast to oxygen and carbon isotopes. *Front. Earth Sci.* **8**, 523073 (2020).

56. T. Arosio, M. Ziehmer, K. Nicolussi, C. Schluechter, A. Thurner, A. Österreicher, P. Nyfeler, M. C. Leuenberger, Alpine Holocene triple tree ring isotope record, PANGAEA (2022); https://doi.pangaea.de/10.1594/PANGAEA.941604.

57. M. M. Ziehmer, K. Nicolussi, C. Schlüchter, M. Leuenberger, Preliminary evaluation of the potential of tree-ring cellulose content as a novel supplementary proxy in dendroclimatology. *Biogeosciences* **15**, 1047–1064 (2018).

58. N. J. Loader, F. A. Street-Perrott, T. J. Daley, P. D. M. Hughes, A. Kimak, T. Levanic, G. Mallon, D. Mauquoy, I. Robertson, T. P. Roland, S. van Bellen, M. M. Ziehmer, M. Leuenberger,

Simultaneous determination of stable carbon, oxygen, and hydrogen isotopes in cellulose. *Anal. Chem.* **87**, 376–380 (2015).

59. M. S. Filot, M. Leuenberger, A. Pazdur, T. Boettger, Rapid online equilibration method to determine the D/H ratios of non-exchangeable hydrogen in cellulose. *Rapid Commun. Mass Spectrom.* **20**, 3337–3344 (2006).

60. M. Leuenberger, To what extent can ice core data contribute to the understanding of plant ecological developments of the past? *Terrestrial Ecol.* **1**, 211–233 (2007).

61. T. B. Coplen, Reporting of stable hydrogen, carbon, and oxygen isotopic abundances (technical report). *Pure Appl. Chem.* **66**, 273–276 (1994).

62. U. Büntgen, T. Kolář, M. Rybníček, E. Koňasová, M. Trnka, A. Ač, P. J. Krusic, J. Esper, K. Treydte, F. Reinig, A. Kirdyanov, F. Herzig, O. Urban, No age trends in oak stable isotopes. *Paleoceanogr. Paleoclimatol.* **35**, e2019PA003831 (2020).

63. G. H. Young, J. C. Demmler, B. E. Gunnarson, A. J. Kirchhefer, N. J. Loader, D. McCarroll, Age trends in tree ring growth and isotopic archives: A case study of *Pinus sylvestris* L. from northwestern Norway. *Global Biogeochem. Cycles* **25**, GB2020 (2011).

64. C. Hartl-Meier, C. Zang, U. L. F. Büntgen, J. A. N. Esper, A. Rothe, A. Göttlein, T. Dirnböck, K. Treydte, Uniform climate sensitivity in tree-ring stable isotopes across species and sites in a mid-latitude temperate forest. *Tree Physiol.* **35**, 4–15 (2015).

65. S. Hangartner, A. Kress, M. Saurer, D. Frank, M. Leuenberger, Methods to merge overlapping tree-ring isotope series to generate multi-centennial chronologies. *Chem. Geol.* **294-295**, 127–134 (2012).

66. T. Arosio, M. Torbenson, T. Bebchuk, A. Kirdyanov, J. Esper, T. Nakatsuka, M. Sano, O. Urban, K. Nicolussi, M. Leuenberger, U. Büntgen, Methodological constrains of tree-ring stable isotope chronologies. *Quat. Sci. Rev.* **340**, 108861 (2024).

67. K. Linnet, Evaluation of regression procedures for methods comparison studies. *Clin. Chem.* **39**, 424–432 (1993).

68. I. Labuhn, V. Daux, M. Pierre, M. Stievenard, O. Girardclos, A. Féron, D. Genty, V. Masson-Delmotte, O. Mestre, Tree age, site and climate controls on tree ring cellulose δ18O: A case study on oak trees from south-western France. *Dendrochronologia* **32**, 78–89 (2014).

69. S. L. Voelker, J. R. Brooks, F. C. Meinzer, J. Roden, A. Pazdur, S. Pawelczyk, P. Hartsough, K. Snyder, L. Plavcová, J. Santrůcek, Reconstructing relative humidity from plant δ18O and δD as deuterium deviations from the global meteoric water line. *Ecol. Appl.* **24**, 960–975 (2014).

70. T. Arosio, U. Büntgen, K. Nicolussi, G. E. Moseley, M. Saurer, T. Pichler, M. P. Smith, E. Gutierrez, L. Andreu-Hayles, I. Hajdas, T. Bebchuk, M. Leuenberger, Tree-ring δ 18O and δ 2H stable isotopes reflect the global meteoric water line. *Front. Earth Sci.* **12**, 1440064 (2024).

71. E. R. Cook, R. Seager, Y. Kushnir, K. R. Briffa, U. Büntgen, D. Frank, P. J. Krusic, W. Tegel, G. van der Schrier, L. Andreu-Hayles, M. Baillie, C. Baittinger, N. Bleicher, N. Bonde, D. Brown, M. Carrer, R. Cooper, K. Čufar, C. Dittmar, J. Esper, C. Griggs, B. Gunnarson, B. Günther, E. Gutierrez, K. Haneca, S. Helama, F. Herzig, K. U. Heussner, J. Hofmann, P. Janda, R. Kontic, N. Köse, T. Kyncl, T. Levanič, H. Linderholm, S. Manning, T. M. Melvin, D. Miles, B. Neuwirth, K. Nicolussi, P. Nola, M. Panayotov, I. Popa, A. Rothe, K. Seftigen, A. Seim, H. Svarva, M. Svoboda, T. Thun, M. Timonen, R. Touchan, V. Trotsiuk, V. Trouet, F. Walder, T. Ważny, R. Wilson, C. Zang, Old World megadroughts and pluvials during the Common Era. *Sci. Adv.* **1**, e1500561 (2015).

72. O. Wetter, C. Pfister, An underestimated record breaking event–Why summer 1540 was likely warmer than 2003. *Clim. Past.* **9**, 41–56 (2013).

73. C. Casty, H. Wanner, J. Luterbacher, J. Esper, R. Böhm, Temperature and precipitation variability in the European Alps since 1500. *Int. J. Climatol.* **25**, 1855–1880 (2005).

74. U. Büntgen, W. Tegel, K. Nicolussi, M. McCormick, D. Frank, V. Trouet, J. O. Kaplan, F. Herzig, K.-U. Heussner, H. Wanner, J. Luterbacher, J. Esper, 2500 years of European climate variability and human susceptibility. *Science* **331**, 578–582 (2011).

75. C. L. Hancock, N. P. McKay, M. P. Erb, D. S. Kaufman, C. R. Routson, R. F. Ivanovic, L. J. Gregoire, P. Valdes, Global synthesis of regional Holocene hydroclimate variability using proxy and model data. *Paleoceanogr. Paleoclimatol.* **38**, e2022PA004597 (2023).

76. A. Mauri, B. A. S. Davis, P. M. Collins, J. O. Kaplan, The climate of Europe during the Holocene: A gridded pollen-based reconstruction and its multi-proxy evaluation. *Quat. Sci. Rev.* **112**, 109–127 (2015).

77. F. He, P. U. Clark, Freshwater forcing of the Atlantic meridional overturning circulation revisited. *Nat. Clim. Change* **12**, 449–454 (2022).

78. F. Joos, R. Spahni, Rates of change in natural and anthropogenic radiative forcing over the past 20,000 years. *Proc. Natl. Acad. Sci. U.S.A.* **105**, 1425–1430 (2008).

79. F. Steinhilber, J. A. Abreu, J. Beer, I. Brunner, M. Christl, H. Fischer, U. Heikkilä, P. W. Kubik, M. Mann, K. G. McCracken, H. Miller, H. Miyahara, H. Oerter, F. Wilhelms, 9,400 years of cosmic radiation and solar activity from ice cores and tree rings. *Proc. Natl. Acad. Sci. U.S.A.* **109**, 5967–5971 (2012).

80. D. C. Salazar-García, O. García-Puchol, "Current thoughts on the neolithisation process of the Western Mediterranean" in *Times of Neolithic Transition along the Western Mediterranean*, D. C. Salazar-García, O. García-Puchol, Eds. (Springer International Publishing, 2017), pp. 1–11; http://link.springer.com/10.1007/978-3-319-52939-4_1.

81. K. Riedhammer, 450 post LBK years in southern Bavaria. *Anthropologie* **53**, 387–398 (2015).

82. A. Hafner, C. Schwörer, Vertical mobility around the high-Alpine Schnidejoch Pass. Indications of Neolithic and Bronze Age pastoralism in the Swiss Alps from paleoecological and archaeological sources. *Quat. Int.* **484**, 3–18 (2018).

83. N. Steuri, M. Milella, F. Martinet, L. Raiteri, S. Szidat, S. Lösch, A. Hafner, First radiocarbon dating of Neolithic stone cist graves from the aosta valley (Italy): Insights into the chronology and burial rites of the Western Alpine region. *Radiocarbon* **65**, 521–538 (2023).

84. M. Besse, *Around the Petit-Chasseur Site in Sion (Valais, Switzerland) and New Approaches to the Bell Beaker Culture: Proceedings of the International Conference (Sion, Switzerland-October 27th–30th 2011)* (Archaeopress, 2014).

85. J. Schibler, The economy and environment of the 4th and 3rd millennia BC in the northern Alpine foreland based on studies of animal bones. *Environ. Archaeol.* **11**, 49–64 (2006).

86. L. Papac, M. Ernée, M. Dobeš, M. Langová, A. B. Rohrlach, F. Aron, G. U. Neumann, M. A. Spyrou, N. Rohland, P. Velemínský, M. Kuna, H. Brzobohatá, B. Culleton, D. Daněček, A. Danielisová, M. Dobisíková, J. Hložek, D. J. Kennett, J. Klementová, M. Kostka, P. Krištuf, M. Kuchařík, J. K. Hlavová, P. Limburský, D. Malyková, L. Mattiello, M. Pecinovská, K. Petriščáková, E. Průchová, P. Stránská, L. Smejtek, J. Špaček, R. Šumberová, O. Švejcar, M. Trefný, M. Vávra, J. Kolář, V. Heyd, J. Krause, R. Pinhasi, D. Reich, S. Schiffels, W. Haak, Dynamic changes in genomic and social structures in third millennium BCE central Europe. *Sci. Adv.* **7**, eabi6941 (2021).

87. M. Bini, G. Zanchetta, A. Perşoiu, R. Cartier, A. Català, I. Cacho, J. R. Dean, F. Di Rita, R. N. Drysdale, M. Finnè, The 4.2 ka BP event in the Mediterranean region: An overview. *Clim. Past* **15**, 555–577 (2019).

88. J. Kleijne, M. Weinelt, J. Müller, Late Neolithic and Chalcolithic maritime resilience? The 4.2 ka BP event and its implications for environments and societies in Northwest Europe. *Environ. Res. Lett.* **15**, 125003 (2020).

89. H.-M. Hu, V. Trouet, C. Spötl, H.-C. Tsai, W.-Y. Chien, W.-H. Sung, V. Michel, J.-Y. Yu, P. Valensi, X. Jiang, F. Duan, Y. Wang, H. S. Mii, Y. M. Chou, M. A. Lone, C. C. Wu, E. Starnini, M. Zunino, T. K. Watanabe, T. Watanabe, H. H. Hsu, G. W. K. Moore, G. Zanchetta, C. Pérez-Mejías, S. Y. Lee, C. C. Shen, Tracking westerly wind directions over Europe since the middle Holocene. *Nat. Commun.* **13**, 7866 (2022).

90. S. F. Warken, J. Fohlmeister, A. Schröder-Ritzrau, S. Constantin, C. Spötl, A. Gerdes, J. Esper, N. Frank, J. Arps, M. Terente, D. F. C. Riechelmann, A. Mangini, D. Scholz, Reconstruction of late Holocene autumn/winter precipitation variability in SW Romania from a high-resolution speleothem trace element record. *Earth Planet. Sci. Lett.* **499**, 122–133 (2018).

91. N. Lončar, M. Bar-Matthews, A. Ayalon, S. Faivre, M. Surić, Holocene climatic conditions in the eastern Adriatic recorded in stalagmites from Strašna peć Cave (Croatia). *Quat. Int.* **508**, 98–106 (2019).

92. K. Nicolussi, G. Weber, G. Patzelt, A. Thurner, A question of time: Extension of the Eastern Alpine Conifer Chronology back to 10 071 b2k. *TRACE* **13**, 69–73 (2015).

93. V. Nagavciuc, M. Ionita, Z. Kern, D. McCarroll, I. Popa, A~ 700 years perspective on the 21st century drying in the eastern part of Europe based on δ18O in tree ring cellulose. *Commun. Earth Environ.* **3**, 277 (2022).

94. I. Labuhn, V. Daux, O. Girardclos, M. Stievenard, M. Pierre, V. Masson-Delmotte, French summer droughts since 1326 CE: A reconstruction based on tree ring cellulose δ18O. *Clim. Past* **12**, 1101–1117 (2016), .

95. U. Büntgen, D. C. Frank, D. Nievergelt, J. Esper, Summer temperature variations in the European Alps, AD 755–2004. *J. Climate* **19**, 5606–5623 (2006).

96. L. Klippel, S. St George, U. Büntgen, P. J. Krusic, J. Esper, Differing pre-industrial cooling trends between tree rings and lower-resolution temperature proxies. *Clim. Past* **16**, 729–742 (2020).
